# Evaluating the impact of the National Health Insurance Fund oncology benefits package and a healthcare workers’ strike on time to cancer treatment initiation in Nairobi County, Kenya: An interrupted time series analysis

**DOI:** 10.1371/journal.pone.0324593

**Published:** 2025-05-22

**Authors:** Robai Gakunga, Anne Korir, Janet Bouttell

**Affiliations:** 1 Independent Research Scientist, Nairobi, Kenya; 2 Kenya Medical Research Institute, Centre of Clinical Research, Nairobi, Kenya; 3 University of Glasgow, School of Health & Wellbeing, Glasgow, United Kingdom; 4 Nottingham University Hospitals NHS Trust, Nottingham, United Kingdom; Purdue University, UNITED STATES OF AMERICA

## Abstract

**Introduction:**

In April 2015, Kenya introduced the National Health Insurance Oncology Benefits Package and its complementary reforms (oncology insurance scheme) to alleviate financial hardship among its members upon a cancer diagnosis. In this study, we hypothesised that the time it took to start treatment would have an impact on health outcomes: the longer patients waited the worse their outcomes would be. We did not have outcomes in the data but we could compute time to treatment initiation (TTI). While assessing the impact of the oncology insurance scheme on TTI, we encountered a substantial sudden increase in average TTI in June 2018 which we needed to explore.

**Methods:**

We conducted our analysis using R, a statistical computing software, for interrupted time series analysis (ITSA) on Nairobi Cancer Registry data to assess the impact of the introduction of the oncology insurance scheme on TTI in days among Nairobi County residents diagnosed with cancer. We calculated the monthly median TTI, resulting in 120 data points (one for each of the 120 months of the observation period - January 1^st^ 2010 to December 31^st^ 2019). Since the oncology insurance scheme was available to the entire Kenyan population, a suitable control group was unavailable. To address this, we used auto regressive integrated moving average (ARIMA) modelling to forecast an expected trend, allowing us to estimate both sudden and gradual changes during April 2015 and June 2018 (intervention months).

**Results:**

After cleaning the data, 7584 (35%) cases of the original 21,464 were left for analysis. Females were more than males at 57.8%. Approximately 65% of the cases with known stage at diagnosis were in stages III and IV. No statistically significant impact was associated with the introduction of oncology insurance scheme; an additional 9.06 days (95% CI: −8.7 to 26.8) and a gradual change of 0.88 days per month (95% CI: −0.11 to 1.88). However, a statistically significant sudden increase in monthly median TTI in June 2018 of 34.6 days (95% CI 15.4 to 53.8) and the gradual change of −1.6 days (95% CI −3.5 to 0.4) per month which was not statistically significant, were associated with a healthcare workers’ strike. We could not accurately analyse case trends from these data because the registry had not completed collating data for the later years (2015–2019).

**Conclusions:**

These results suggest that the oncology insurance scheme may not have reduced average TTI for the cancer patients as we had hypothesized. However, a healthcare workers’ strike (based on corroboration with findings from the 2018 Kenya Household Health Expenditure and Utilization Survey), increased the average TTI among these patients. Data science techniques and ITSAs using cancer registry data is a cost-effective method to answer important population-level research questions in resource-limited settings.

## Introduction

The cost of cancer care in Kenya is high and can be prohibitively expensive for many. Direct medical costs like consultation fees, drug costs, and non-medical costs like travel, lodging and supportive care can cause substantial financial hardship to patients and their families especially when patients lack financial stability or insurance [1]. A recent study in Kenya found that approximately 50% of breast cancer patients forego or are deterred from seeking care due to inadequate insurance cover or inability to pay out-of-pocket [2]. Financial hardship may also lengthen time to treatment initiation (TTI) (the time between disease diagnosis and the beginning of definitive cancer treatment), as patients and their families consolidate required finances. Treatment delays are associated with significant tumour growth and a greater likelihood of increasing the clinical cancer stage and recurrence, as well as reducing quality of life and survival [3–10]. In addition to poorer health outcomes, higher clinical cancer stages at diagnosis are more costly to manage [11]. Minimizing TTI is a fundamental goal in cancer care and reducing financial barriers to care may encourage more cancer patients to promptly seek care and to comply with healthcare providers’ recommendations for better outcomes.

The Kenyan government introduced the National Health Insurance Fund Oncology Benefits Package together with its complementary reforms (oncology insurance scheme), in April 2015. The scheme covered consultation, laboratory investigations, drugs and dispensation, radiology, surgical procedures, chemotherapy and radiotherapy [12,13]. The aim was to provide protection against out-of-pocket payments and impoverishment for the scheme’s members in the event of a cancer diagnosis [14]. The Kenya National Health Insurance Fund (NHIF) (now replaced by the Social Health Authority) was a state corporation where members received health services in return for contributions based on their employment status [14,15].

Financing of healthcare in Kenya comes from taxation, out of pocket spending, user fees, funding from donors, insurance (public and private) and charities [16]. Funding from donors has been reducing over time as the Kenyan government gradually transitions from donor dependency to domestic public financing [17,18]. The Kenyan government’s health expenditure as a percentage of total expenditure has increased over time from 4.8 percent in 2008/2009–11 percent in 2023/2024 [19] although it falls short of the 15 percent commitment in the 2001 Abuja declaration [20]. Available data show that out of pocket expenditure as a percentage of total health expenditure generally remained constant at approximately 25 percent from 2008 to 2016 [15].

Healthcare insurance aims to cushion members from exorbitant out of pocket spending and poverty in case of illness. Individuals pool funds which are then managed by a third party (government, employers or insurance companies) that pays for members’ healthcare costs. The 2018 Kenya Household Health Expenditure and Utilization Survey (KHHEUS) recorded that 19.9 percent (of 47.8 million Kenyans) had health insurance coverage with some respondents having more than one policy [12]. This was an improvement on historic figures (17.1 percent in 2013, 10 percent in both 2003 and 2007). Catastrophic health expenditure occurs when out-of-pocket spending for health care exceeds “a certain proportion of a household’s income with the consequence that households suffer the burden of disease” [14]. Kenya uses two thresholds for catastrophic health expenditure: out of pocket spending on healthcare exceeds 10 percent of a household’s total expenditure or 40 percent of spending of non-food expenditure. In the 2018 KHHEUS, 8 percent of households were experiencing catastrophic health expenditure using the 10 percent of total expenditure threshold, and 4.9 percent of households using the 40 percent of non-food expenditure threshold. These figures were lower than the 2013 equivalents of 12.7 percent and 6.2 percent respectively (S1 Fig) [14]. The main coping mechanism that households without insurance or readily available cash use to manage healthcare costs is money given by family and friends. Other coping mechanisms include borrowing, disposal of assets and fundraising among well-wishers [21]. A study at three leading cancer treatment centers in Kenya (Kenyatta National Hospital, Aga Khan University Hospital and Moi Teaching and Referral Hospital) found that 81% of cancer patients are exposed to borrowing for their treatment [22].

Anticancer drugs are expensive, and their cost continues to increase as new drugs come to the market. A Kenyan study published in 2018 estimated costs of up to USD 12,000 per patient for breast and cervical cancer care in the first year of treatment [11].

Reducing the financial burden associated with healthcare can lead to increased use of health services and improved health outcomes, particularly among low-income households [23,24]. In 2012 Chuma and others evaluated the impact of out-of-pocket payments on healthcare utilization in Kenya using the 2007 KHHEUS data. They found that on average, Kenyan households spend a tenth of their budget on healthcare and poorer populations spend disproportionately higher fractions of their budget. They recommended the need for health system reforms so that all citizens are protected from financial risk in the event of illness. With some reforms, the trend in catastrophic health expenditure in Kenya reduced significantly [25].

Evaluations of NHIF’s performance have mainly been qualitative, conducted by interviewing health financing stakeholders at national and county levels, facility managers, frontline providers, NHIF members, and document reviews focusing on the reforms [14]. These studies revealed some performance gaps and the need for policymakers at the NHIF and government (both national and county) to rework design and implementation strategies, to align them with strategic purchasing [14]. Quantitative research to evaluate the impact of the NHIF intervention on health outcomes is lacking. The aim of this study was to quantitatively evaluate the impact of Kenya’s oncology insurance scheme, on treatment outcomes among residents of Nairobi County.

## Methods

### Study design

This was an observational study using interrupted time series analysis (ITSA) with autoregressive integrated moving average (ARIMA) modelling.

### Study setting

The setting was Nairobi County, one of the 47 counties in Kenya and the most populous. It hosts Kenya’s capital, the Nairobi city. Cancer surveillance in Nairobi County began in 2001 at the Nairobi Cancer Registry (The Registry) which is based at the Center of Clinical Research at the Kenya Medical Research Institute.

### Data description

We utilized de-identified, password-protected cancer incidence data from The Registry, for the period covering January 1, 2010, to December 31, 2019, as of April 13, 2022. Data were received in MS Excel format and included the following variables; age, sex, incidence date, basis of diagnosis, primary site, morphology, surgery (whether surgery was provided or not), surgery date, radiotherapy (whether radiotherapy was provided or not), radiotherapy date, chemotherapy (whether chemotherapy was provided or not), chemotherapy date, hormonotherapy (whether hormonotherapy was provided or not), hormonotherapy date, immunotherapy (whether immunotherapy was provided or not), immunotherapy date and registration number (19 variables in total). Data collation in the later years (2015–2019) was incomplete. Despite this incompleteness, we had sufficient data to estimate the monthly median TTI, assuming the missing data were missing at random. We were unable to determine the exact number of cases in the later years, which prevented us from analyzing the trend. Registries take a long time to compile data for all cases, resulting in an inevitable lag period.

### Data collection and data quality

The Registry is population based and covers an urban population of approximately 4.4 million (2019) [25] and a geographical area of 695.1 km2 [26]. Together with the Eldoret Cancer Registry in the northern part of Kenya, The Registry’s data are used for providing national estimates for the World Health Organization’s International Agency for Research on Cancer’s GLOBOCAN report. The Registry’s operations are based on the Africa Cancer Registries Network (AFCRN)’s standard operating procedures manual [26] which are mostly derived from the International Agency for Research on Cancer (IARC)’s Global Initiative for Cancer Registry Development and IARC’s internationally used guidelines. Consistency of recording of data (like in cancer registry data) is a strength for conducting time series analysis where changes in definitions and/or measurement of variables may introduce bias [27]. The Registry’s data are collected manually on paper although there are efforts to implement more cost effective and efficient electronic data collection methods. The Registry is a member of the AFCRN, which accepts registries that meet high-quality standards and cover at least 70% of their target population. Data from The Registry were included in volume XII of the IARC/International Association of Cancer Registries’ publication, Cancer Incidence in Five Continents (CI5) [28], which only admits data of good quality.

### Outcome measure

The outcome measure for the ITSA was TTI. We calculated TTI as the difference in number of days between the date of the first definitive cancer treatment (surgery, chemotherapy, radiotherapy, hormonotherapy and immunotherapy) received by the patients and the date of diagnosis (incidence date). There were 10,329 (48.1%) cases with missing TTI values. These were excluded from the analysis as explained in S2 File.

We presumed that TTI is an objective measure, and a health system performance indicator, where longer TTIs can be associated with worse outcomes [3–10]. We aggregated the TTI values into monthly medians to get 120 data points (each one for each of the 120 months in the observation period) with 63 data points before the intervention month (April 2015) and 56 data points post intervention. The median as a measure of central tendency for each month was appropriate because of the possibility of having distant outliers in cancer registry data. Outliers may distort ARIMA/SARIMA modelling in the ITSA. The step-by-step process of data cleaning and data manipulation is available from S2 File.

### Sample size

We analysed a total of 120 time series data points. The April 2015 data point was the 64^th^ in the series providing for balanced study periods around the intervention which adds to the power of the analysis [29]. There is no formal basis of selection of sample sizes for ITSAs, however, we considered this sample size adequate because it was more than the minimum recommended by the Effective Practice and Organisation of Care (EPOC) (three on each side of the intervention) [30] and by Box and Jenkins (minimum of 50 data points) [31]. Schaefer and others’ view is that the more complex the time series is, the more the time points required. They are of the opinion that in the presence of seasonality, “there is need for more time points to capture seasonal effects and to allow for seasonal differencing” [31]. Zhang and others’ opinion is that time series analyses’ statistical power increases with increasing number of data points [29].

### Assumptions for the Interrupted Time Series Analysis (ITSA)

We assumed that all other factors that would affect TTI; economic, social, logistical, clinical complexity of the diseases and health system factors remained constant around the intervention months. ITSA is only valid if the intervention being evaluated is the only event that changed at the demarcated time. Another important assumption we made was that the data we received from The Registry was of good quality, basing this on The Registry’s contributions and memberships to regionally and internationally recognized cancer registry bodies.

### Data analysis

We used R, a statistical computing software (with the forecast, astsa, dplyr, zoo, ggplot and tseries packages) to carry out ITSA with monthly median TTI as the outcome measure to evaluate the impact of the oncology insurance scheme (introduced in April 2015) and an unexpected increase in monthly median TTI in June 2018 (The exact location of the 2018 increase was determined as the month of June by closely reviewing the time series data).

#### Interrupted time series analysis using autoregressive integrated moving average (ARIMA) modelling.

ITSA is a “powerful quasi-experimental design” [32] that may be used to evaluate the impact of policy interventions like the introduction of the oncology insurance scheme on health outcomes.

Interrupted time series designs are very appealing to audiences because of the ability to present the outcome variation over time in clear graphics, and reveal any unintended or unexpected consequences of interventions [32]. Importantly, ITSA designs can assess the impact of policies that cannot be otherwise evaluated due to ethical reasons or resource constraints, and can postulate causality in lieu of randomized controlled trials.

**Fig 1:**
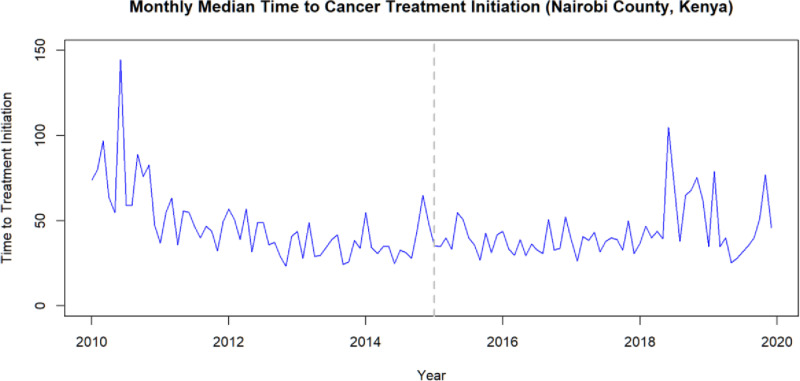
Monthly median time to cancer treatment initiation for Nairobi County, Kenya; Intervention line - April 2015 NHIF oncology benefits package introduction, Nairobi Cancer Registry Data (2010–2019).

We hypothesized that the oncology insurance scheme decreased TTI among cancer patients in Nairobi County with a step (sudden permanent shift in the outcome) and/or a ramp (gradual change in outcome) during or from the month of intervention. First, we plotted a graph to visualize and understand our time series data (Fig 1).

This graph revealed an initial downward trend of monthly median TTI from 2010 to 2014, followed by a slight upward movement and then another decline before a noticeable sudden increase in 2018. To better visualize trends and seasonality, we decomposed the time series data, which confirmed the presence of both trend and seasonal patterns. The decomposition chart for the time series data is available from S3 Fig.

Second, we checked for stationarity and autocorrelation in our time series. The assumptions for generating an ARIMA or its extension SARIMA (Seasonal Autoregressive Integrated Moving Average) model that includes a seasonal component, are that data should be stationary which means that the statistical properties of the data (mean, variance and covariance) remain constant with time [31], and data should not be autocorrelated, where “observations in a time series are correlated with observations at previous time points hence violating their independent distribution” [31]. Our data were not stationary (Augmented Dickey-Fuller test - p-value: 0.4533) and they were autocorrelated based on ACF (Autocorrelation Function) and PACF (Partial Autocorrelation Function) tests. They required differencing - a technique used to make a time series stationary by removing trends and systematic patterns.

We applied the auto.arima () function in the *forecast package* which automates the selection of the best ARIMA model based on provided data. The best model was ARIMA (0,1,4). We found that it was correctly specified after testing it for appropriateness and accuracy using the Ljung-Box test using the Box.test () function (p-value was 0.9935 at 0.05 level of significance).

Because of the seasonality visualised in our data, we included a seasonality component in the model SARIMA (0,1,4) (0,1,1) [12]. Another Ljung-Box test indicated that this model was correctly specified (p-value was 0.9745 at 0.05 level of significance) and could be used to forecast a counterfactual by fitting the SARIMA model to pre-intervention data, in order to assess deviations from this forecast in the post-intervention period. The ARIMA/SARIMA modelling process includes exploratory analysis, model fitting, and diagnostic evaluation. The details and R codes are provided in S4 File. A power calculation for this model using the forecast package in R, based on 1,000 simulations, p-values < 0.05, and an effect size of 7 days monthly median TTI was 71%. (R codes for this power calculation are available from S5 File).

#### Impact evaluations.

**Fig 2:**
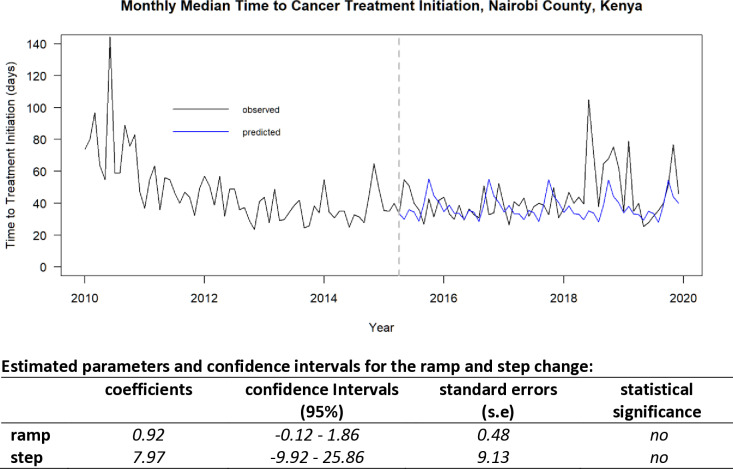
Interrupted time series analysis; the National Health Insurance Fund (NHIF) oncology package Intervention (April 2015) for Nairobi County, Kenya. (Nairobi Cancer Registry data; 2010–2019).

**Fig 3:**
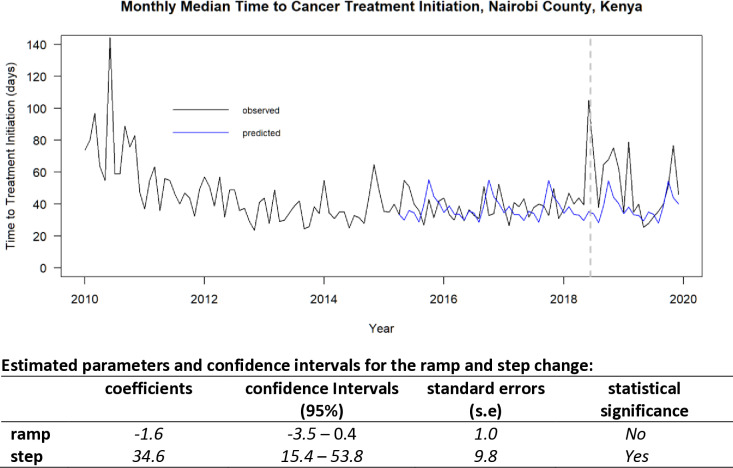
interrupted time series analysis: Monthly Median Time to Treatment Initiation. **Impact of interruption due to a healthcare workers’ strike in June 2018 for Nairobi County, Kenya.** (Nairobi Cancer Registry data; 2010–2019).

We plotted the counterfactual series (expected trend) for the post intervention period and estimated the regression coefficients and confidence intervals to test the statistical significance of the step and ramp changes arising from the introduction of (i) the oncology insurance scheme in the time series (Fig 2) and (ii) the June 2018 increase in monthly median TTI in the time series (Fig 3).

### Ethical considerations

This study was approved by the institutional review board of the Kenya Medical Research Institute (SERU Protocol No. 3262) through the Nairobi Cancer Registry as part of the registry’s activities. Cancer registry data are sensitive data. Before they were provided for analysis, they were de-identified and the document was password protected.

## Results

### Demographics and clinical characteristics of cancer patients in Nairobi County, Kenya

The total number of cases received for this analysis was 21,464. After data cleaning (addressing missing values and data entry errors) we retained 7,584 cases (35%). Table 1 shows the demographics, clinical characteristics and the number of cancer cases by year of diagnosis for Nairobi County (n = 7,584) as on April 13^th^ 2022. Approximately 58% of the cancer cases were female, 42% were male and no intersex were recorded. About 7% were less than 18 years old. The stage at diagnosis was unknown for 62% of the cases. Of those with known diagnosis (n = 2,558), stages III and IV were most prevalent (34% and 31% respectively). We were not able to establish a trend by year of diagnosis because in the later years (2015–2019) data collation at the Registry was incomplete. The most common treatments received among those included in this analysis were chemotherapy (42%), followed by radiotherapy (31%) and surgery (24%).

**Table 1:** Demographics, clinical characteristics and number of cases by year of diagnosis for incident cancer cases in Nairobi Kenya (2010–2019) of the cleaned dataset (n = 7584).

	Number	Percent (%)
**Gender**		
Female	*4383*	*57.8*
Male	*3201*	*42.2*
**Age group (years)**		
<18	*535*	*7.1*
19–30	*499*	*6.6*
31–40	*1082*	*14.3*
41–50	*1535*	*20.2*
51–60	*1688*	*22.3*
61–70	*1335*	*17.6*
70 +	*893*	*11.8*
**Cancer stage**		
Stage I	*222*	*2.9*
Stage II	*690*	*9.1*
Stage III	*864*	*11.4*
Stage IV	*782*	*10.3*
Unknown	*5026*	*62.2*
**Year of diagnosis**		
2010	*691*	*9.1*
2011	*950*	*12.5*
2012	*925*	*12.2*
2013	*915*	*12*
2014	*955*	*12.6*
2015	*744*	*9.8 (incomplete data)*
2016	*744*	*9.8 (incomplete data)*
2017	*637*	*8.4 (incomplete data)*
2018	*591*	*7.8 (incomplete data)*
2019	*432*	*5.7 (incomplete data)*
**Total number of treatments received across all cancers**		
Surgery	*2563*	*24.1*
Chemotherapy	*4451*	*41.8*
Radiotherapy	*3327*	*31.2*
Immunotherapy	*30*	*0.3*
Hormonotherapy	*282*	*2.6*

### Impact of the oncology insurance scheme on time to treatment initiation among Nairobi County residents

There was no statistically significant difference between the observed and predicted values (step change 7.97 days (95% CI −9.92 to 25.86), ramp change 0.92 days per month (95% CI: −0.12 to 1.86)). Fig 2 shows that the observed and the predicted monthly median TTI trends. Seasonality remained similar but with higher fluctuations for the predicted values until 2018 when there was a sudden increase in the observed monthly median TTI value. This led us to investigate the potential cause of the TTI increase in 2018.

### Investigating the sudden increase in time to treatment initiation in 2018

Although this was not part of the aims for this study, we explored the sudden increase in monthly median TTI in 2018 using a similar approach as the one we used for the introduction of oncology insurance scheme. An increase in monthly median TTI (step change) in June 2018 of 34.6 days (95% CI 15.4 to 53.8) was statistically significant at the 95% confidence level and a gradual decrease (ramp change) of −1.6 days (95% CI −3.5 to 0.4) per month was not statistically significant at 95% confidence level (Fig 3).

#### What could have caused the sudden increase in the average time to treatment initiation in June 2018?.

**Fig 4:**
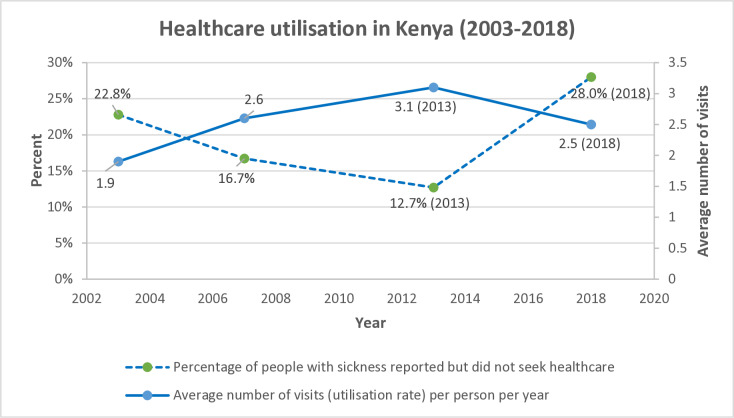
Healthcare utilization Trends in Kenya. Redrawn, with modification, from Ministry of Health, Government of Kenya 2018 Kenya Household Health Expenditure and Utilization Survey (2019). Kenya National Bureau of Statistics. Kenya Household and Health Expenditure and Utilization Survey (KHHEUS) 2018 [Internet]. [cited 2023 Apr 13]. Available from: https://statistics.knbs.or.ke/nada/index.php/catalog/95/related-materials.

We searched literature in key government documents and made some inquiries from key healthcare personnel. According to the 2018 Kenya Household Health Expenditure and Utilization Survey (KHHEUS), the proportion of individuals reporting sickness was 19% in both 2013 and 2018 (we took these as reference values). The percentage of people with some sickness reported but did not seek health care was 12.7% in 2013 and 28% in 2018 (a negative effect occurred) and the average number of visits per person per year was 3.1 and 2.5 in 2013 and 2018 respectively (a negative effect occurred) (Fig 4) There was a general negative impact on healthcare indicators in 2018 which the ministry of health attributed to a healthcare workers’ strike [14].

### Sensitivity analysis

#### Variation of the pre-intervention time frames (oncology insurance scheme).

**Fig 5:**
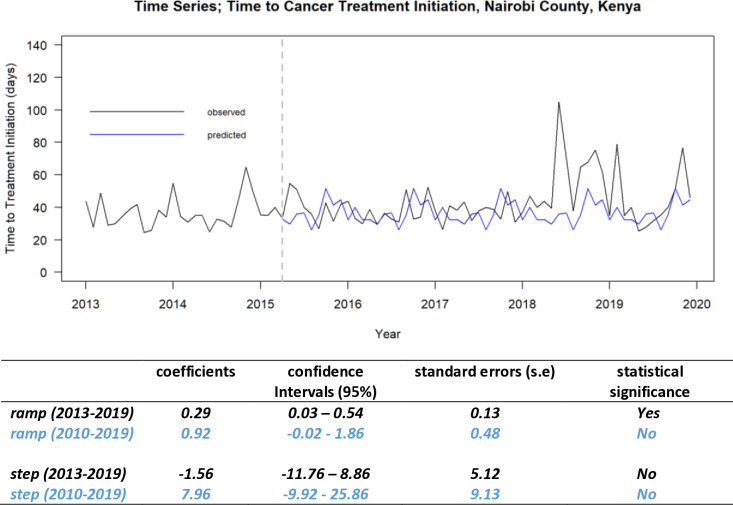
Interrupted time series analysis: introduction of the NHIF oncology benefits package (April 2015) - Forecasting counterfactual from March 2015. Nairobi County, Kenya (2013–2019/2010–2019 Nairobi Cancer Registry data).

We were interested in the impact on the result if the volatile period pre 2013 was excluded from the analysis. Fig 5 is the time series plot for the reduced pre-intervention time frame (analysis from the 2013–2019 time series subset). We included the 2010–2019 data set intervention effect parameters in this chart for comparison**.**

The ramp change of 0.29 days (95% CI 0.03 to 0.54) per month was statistically significant in the 2013–2019 data set but not in the 2010–2019 data set. This implies that unlike in the 2010–2019 data set model, where there was no apparent change, the 2013–2019 data set model had a 0.29 day increase per month in monthly median TTI after the introduction of the oncology insurance scheme. Standard errors were smaller with the 2013–2019 data set.

#### Variation of the pre-intervention time frames (healthcare workers’ strike).

We also carried out sensitivity analyses for the healthcare workers’ strike disruption of monthly median TTI by varying the data time frames and summarised the results in table 2.

**Table 2:** Sensitivity analysis; varying time frames in the interrupted time series analyses of median monthly time to cancer treatment initiation of 2010–2019 Nairobi Cancer Registry data; Healthcare workers strike (2018) for Nairobi County - Forecasting counterfactual from March 2015.

	Coefficients	Confidence intervals (95%)	Standard errors (s.e)	Statistical significance
**Ramp (2010–2019)**	−*1.6*	−*3.5–0.4*	*1.0*	*No*
**Ramp (2013–2019)**	−*1.8*	−*2.8 –* −*0.9*	*0.5*	*Yes*
**Step (2010–2019)**	*34.6*	*15.4–53.8*	*9.8*	*Yes*
**Step (2013–2019)**	*33.4*	*22.3–44.5*	*5.6*	*Yes*

The ramp change was statistically significant in 2013–2019 data for the healthcare workers’ strike: −1.8 (95% CI −2.8 to −0.9) but not in the 2010–2019 data. The step changes were both statistically significant at an approximate increase of 33–34 days monthly median TTI. This implies that the 2018 sudden increase was an immediate large impact (step change) followed by gradual reduction over time (ramp change) of 1.8 days per month for the 2013–2019 data while there was no gradual change for 2010–2019 data. Standard errors were smaller with the 2013–2019 data set.

(*R codes and details of data analysis are available from* S4 File).

## Discussion

This study evaluated the impact of introducing the oncology insurance scheme on cancer care outcomes using time to treatment initiation (TTI) as a proxy for treatment outcomes. Our theory of change was that while costs of care remain a key barrier and hence a determinant of treatment outcomes, interventions to minimize costs to the patient, would reduce TTI and lead to better outcomes. Findings from this study provide valuable insights on the impact of the oncology insurance scheme introduced in Kenya in April 2015 to inform further policy decisions. They also contribute to the growing use of quasi-experimental designs in public health policy evaluation in resource limited settings.

Our findings suggest that there was no statistically significant effect, neither increase nor decrease, in average TTI among cancer patients resident in Nairobi County following the introduction of the oncology insurance scheme in April 2015; step (sudden and sustained) change: 9.06 days (95% CI: −8.7 to 26.8) and ramp (gradual) change: 0.88 days per month (95% CI: −0.11 to 1.88). Although this analysis did not find a statistically significant change in average TTI among these patients, this does not imply that the introduction of the oncology insurance scheme was without benefit. The scheme may have facilitated patient access to better, higher-cost treatment regimens or improved treatment completion by making it more affordable for patients and their families. The new public insurance in Kenya, by the Social Health Authority fully covers diagnostics as well as treatments for chronic illnesses like cancer and this has greater potential to change TTI in the population. Despite the risk of using TTI derived from routinely collected patient data as a proxy for health outcomes to assess the impact of a large-scale health policy intervention, the cost-effectiveness and practicality of the process remains substantial.

This study analysed a population that was able to cope with financial burdens arising from healthcare costs through insurance, by disposing off their assets, borrowing and fundraising [21,22] — at least enough to start treatment. However, there were 10,329 (48.1%) patients who did not have any treatment start dates registered by The Registry. What happened to them? Did they fail to receive care? Did they seek care from facilities that The Registry did not reach. Insights from this discussion may support the efforts to refine the Social Health Authority implementation strategies and to establish/strengthen disease registries to improve surveillance.

### Impact of healthcare workers’ strikes on treatment outcomes

There was a statistically significant increase in monthly median TTI of 34.6 days (95% CI: 15.4 to 53.8) in June 2018. In the 2018 KHHEUS report, the percentage of people with some sickness reported who did not seek healthcare increased in 2018 and the average number of visits per person per year decreased in 2018 [14]. The ministry of health attributed these negative changes in the healthcare system to a healthcare workers’ strike. We found it justifiable to attribute the increase in TTI in 2018 to the same healthcare workers’ strike.

Irimu and others investigated the effect of a 2017 doctors’ strike on healthcare utilization in Kenya using the number of admissions per month as a proxy. Their study’s data was from 13 first referral county (public) hospitals. They found substantial decreases in the number of admissions at these hospitals during the months with doctors’ strikes [33]. Interestingly, our time series analysis did not show significant trend changes in the charts in 2017. A possible explanation for this is that Nairobi County hosts the level six Kenyatta National Hospital (the only public cancer treatment hospital at the time) whose cancer treatment units remained functional despite the 2017 nationwide healthcare workers’ strike that affected public hospitals.

### Sensitivity analysis of the interrupted time series analysis/autoregressive integrated moving average modelling

Sensitivity analysis to assess the interventions impact for different pre-intervention time periods (the 2013–2019 time series versus the 2010–2019 time series), found that the shorter pre-intervention period elicited statistical significance where the longer pre-intervention period did not. The standard errors were consistently smaller with the shorter pre-intervention period and therefore an apparent higher precision than the longer pre-intervention period dataset (Fig 5 and Table 2). Zhang and others consider longer pre-intervention periods superior because of possible larger sample size, better statistical power and model stability [29]. It is crucial to carefully consider the limitations and potential biases introduced by shorter time periods and interpret the findings with caution. Researchers and analysts should also consider that interventional effects may be statistically significant but not necessarily of public health significance.

### Demographics, trends and clinical characteristics of incident cancer cases in Nairobi, Kenya

On analysing the cleaned dataset (n = 7,584), there were more females than males (57.8% vs 42.2%). This ratio was quite similar to the ratio reported in the most recent Nairobi Cancer Registry report (2009–2013) which had a total of 10,344 cases (57.7% females,42.3% males). In this analysis, approximately 65% of the cases with known stage at diagnosis were in the late stages (i.e., III and IV) which approximates the 67% that was reported in the 2021 Kenya National Breast Cancer Screening Action Plan [34]. The similarities of important statistical parameters from our analysis with government parameters add validity to the data science techniques used to clean and manipulate the raw data for our analysis. We could not accurately analyse incidence trends because of incomplete collation of the data in the later years (2015–2019). The Registry’s data collection activities were interrupted by movement and access restrictions necessitated by the Covid 19 pandemic, in addition to usual financial constraints. It would be interesting to establish the absolute number of patients who sought care after April 2015, and if the analysis of complete data would yield similar or different results.

### Strengths of this study

We used ITSA to leverage its strength of low cost yet powerful design to evaluate the impact of a policy intervention on health outcomes using routinely collected cancer registry data. Periodic fluctuations, that were seasonal, and secular trends were accounted for using ARIMA models [29] and counterfactuals were modelled and plotted to represent expected trends which were used for comparison with the observed trends. ITSA offers a significant advantage over simple pre-post comparisons, such as t-tests of average values before and after the intervention. Unlike t-tests, ITSAs account for pre-existing trends and seasonal variations, providing a more robust assessment of the intervention effects [24].

The Nairobi Cancer Registry has a database since 2001. We leveraged this quantity of data to accumulate 120 observations of monthly median TTI over a 10-year period. (63 time points before and 56 time points after the intervention) cost effectively. The minimum suggested data points especially when dealing with seasonal data is fifty [27] and we considered the 71% power calculated from 1000 simulations from our SARIMA model (p value – 0.05) for an effect size of 7 days to be reasonable given the constraints of real world data.

### Limitations of this study

A potential limitation of this study was that the Nairobi Cancer Registry had not completed data collation for later years (2015–2019) by the time we received the data for this study. We have no reason to believe that the missing data introduced bias into the analysis, though we cannot be entirely certain. Where there was any doubt about the reliability of the data, for example if the date of diagnosis was later than the date of first treatment, we excluded the case from the analysis.

Using real world data for research is less costly than using alternative methods but the disadvantage is that these data are not collected for specific research questions and may have quality issues. In spite of this, data science techniques and careful handling of missing data and other errors can refine the data to make them useable for answering many research questions.

This study’s design could have been stronger if we had a suitable control. This was not possible because the oncology insurance scheme was implemented countrywide and at the same time point. Having a control in ITSA may separate intervention effects from other effects that may happen at the same time point [27] to reduce confounding. Additionally, a prospective study with randomization would have been preferable, as randomized controlled trials are the gold standard for causal evaluations. However, our intervention was historical, and the associated costs would have been significantly higher.

Several factors other than costs of healthcare may influence TTI. These factors include the clinical characteristics of cancers that may require additional tests and consultations, decision-making processes involving patients, families, and healthcare providers that could contribute to treatment delays, and healthcare system factors such as the availability of equipment, supplies, staffing, and insurance coverage for diagnostic procedures. We assumed that these factors remained constant throughout the intervention periods.

## Conclusions and recommendations

We have demonstrated the possibility of using data science techniques to organize registry data for ITSAs, to evaluate population level interventions. This study adds to the increasing application of quasi-experimental designs in public health policy evaluations.

Stratifying the analysis by sex, cancer type, age group, and stage at diagnosis could offer valuable insights into variations, helping to identify subpopulations that may require greater support when accessing cancer care. However, it is important to note that the dataset may not have enough observations to support this level of stratification. Further research should examine how the intervention affects prescribing patterns and treatment completion.

## Supporting information

S1 Fig Catastrophic health expenditure trend in Kenya. (PDF)

S2 File Supporting Information. Step-by-step process of data cleaning and data manipulation.
(PDF)

S3 Fig Decomposition of time series of monthly median Time to Treatment Initiation. Nairobi County cancer registry data (2010–2019).
(PDF)

S4 File Supporting Information. Details of data analysis and R codes.
(PDF)

S5 File Supporting Information. R codes for model power estimation.
(PDF)
