# Supplementary material for: Evaluating the impact of the National Health Insurance Fund oncology benefits package and a healthcare workers’ strike on time to cancer treatment initiation in Nairobi County, Kenya: An interrupted time series analysis
Source: PLoS One. 2025 May 22;20(5):e0324593. doi: 10.1371/journal.pone.0324593 (PMC12097610; doi:10.1371/journal.pone.0324593)
Supplement: S1 Fig — (PDF) [file pone.0324593.s001.pdf]

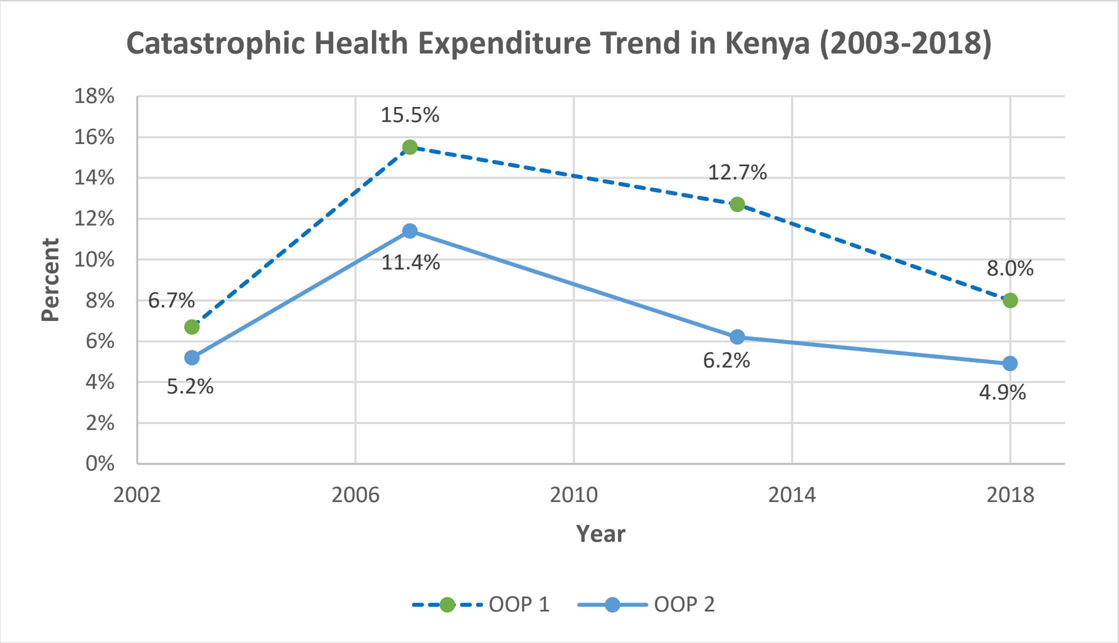

Fig 1. Catastrophic health expenditure trend in Kenya.
OOP 1: Out of pocket expenditure as a share of total expenditure (10% threshold), ***OOP 2***: Out of pocket expenditure as a share of total non-food expenditure (40% threshold). Redrawn, with modification, from Ministry of Health, Government of Kenya 2018 Kenya Household Health Expenditure and Utilization Survey (2019) (1)

References:

1. Kenya - KENYA HOUSEHOLD AND HEALTH EXPENDITURE AND UTILIZATION SURVEY (KHHEUS) 2018 [Internet]. [cited 2023 Apr 13]. Available from: https://statistics.knbs.or.ke/nada/index.php/catalog/95/related-materials
