# Supplementary material for: Evaluating the impact of the National Health Insurance Fund oncology benefits package and a healthcare workers’ strike on time to cancer treatment initiation in Nairobi County, Kenya: An interrupted time series analysis
Source: PLoS One. 2025 May 22;20(5):e0324593. doi: 10.1371/journal.pone.0324593 (PMC12097610; doi:10.1371/journal.pone.0324593)
Supplement: S2 File — Step-by-step process of data cleaning and data manipulation. (PDF) [file pone.0324593.s002.pdf]

S2 Supporting Information

Data cleaning and data manipulation

We imported data from MS Excel into R, explored the data and converted the variables into formats that were useable for our analysis. We created a column for TTI (time to treatment initiation) in days to represent the difference in days between the incidence date and the earliest treatment start date among the five definitive treatment options; surgery, chemotherapy, radiotherapy, immunotherapy and hormonotherapy ***[R Codes: Resource 2, Section A].*** In this column, there were 10,329 "NAs" (missing values); 48.1% of the total 21,464 cases received. The cases with missing TTIs were not valid for our analysis. They had no treatment dates recorded.

Handling Missing Values

There was a big fraction (48.1%) of missing TTI values in these data. The option of imputation to resolve this problem was not going to be realistic because cases with recorded treatment start dates may have been easier to abstract than those without recorded treatment start dates due to varying access to data at data source facilities. There is no standard way of determining and categorising ease of case finding at data source facilities. Missingness was at random and imputation without other appropriate variables, especially important ones like measurements of level of data access at source facilities, may have given misleading results. For this reason, complete case analysis (CCA), where only cases with treatment start dates were included, was the appropriate method of dealing with missingness in the outcome variable (TTI).

To check on the validity of using CCA, we compared the distribution of key variables for all cases in the dataset, those with, and those without missing data. We compared the distribution of percent counts of TTI variable (all cases), TTI variable with "NAs" (missing values) and TTI variable with values, based on variables that are important determinants of TTI or sub groups in the population; age-group, sex, stage at diagnosis and year of diagnosis using histograms in MS Excel (Figure 1) after extracting data from R ***[R Codes: Resource 2, Section B].***

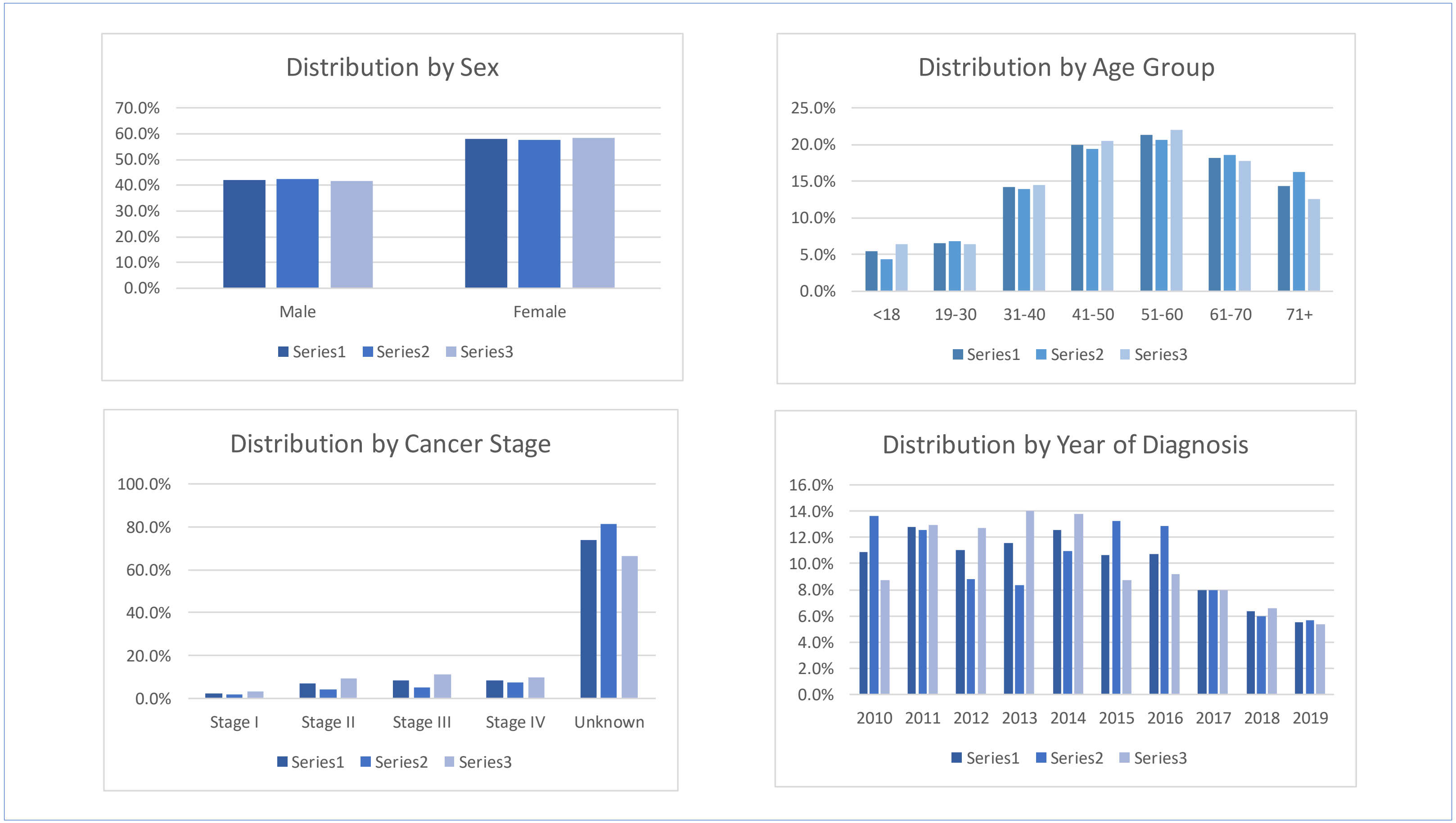

Fig 1. Understanding missingness using histograms
Comparing distribution of time to treatment initiation (TTIs) for the entire dataset (N= 21464) (Series 1), the cases with missing TTIs (n= 10329) (Series 2) and the cases with TTIs (n=11135) (Series 3).
Comparison based on other variables that are important determinants of TTI or population sub groups; sex, age-group, stage at diagnosis and year of diagnosis. Data from the Nairobi Cancer Registry (2010-2019).

Distributions by "sex" and "age group" were similar, but those by "cancer stage" and "year of diagnosis" were somewhat varied in some of the categories. For example, missing values among stage III cancer cases were lowest while missing values in the unknown stage category were highest. Missing values were highest in 2010, 2015 and 2016. Valid cases (those with a TTI) were highest in 2012, 2013 and 2014.

Generally, there was reasonable similarity across categories hence we held our assumption of missingness at random. We found it reasonable to exclude the cases with missing TTI with the assumption that this would not introduce significant bias and there wouldn't be loss of good representation in key sub groups.

We then used a histogram to visualize the frequencies of TTI for the remaining cases (11,135 cases). This revealed unexpected negative TTI values (Figure 2). Approximately 30% of the 11,135 cases had negative TTI values ***[R Codes: Resource 2, Section C]***

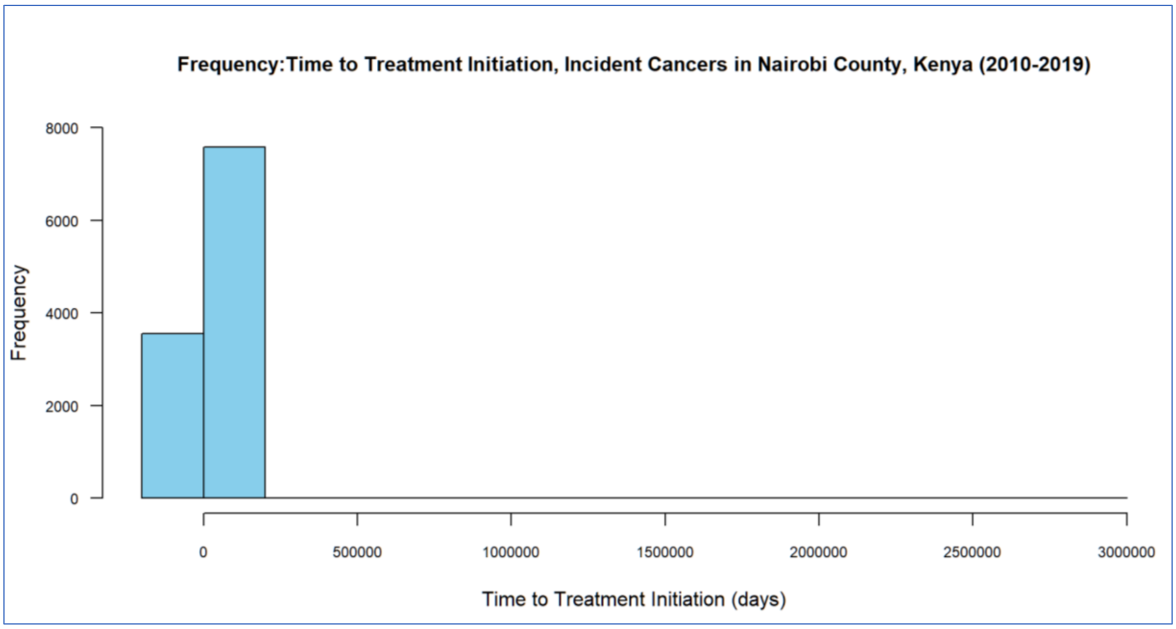

Fig 2. Frequency chart of Time to Treatment Initiation for Incident Cancer Cases in Nairobi County, Kenya (2010-2019).
Data from the Nairobi Cancer Registry.

The reason for negative TTI values in the dataset was possible errors in recording treatment start dates or there were pending updates on incidence dates after abstracting new data. The AFCRN standard operating procedures manual recommends updating of incidence dates as new information becomes available. There is a chronological order by which incidence dates should be recorded. The highest priority is given to the biopsy date of the first histological or cytological

confirmation and the lowest priority is given to the date of death if the tumour was diagnosed at autopsy (*R**esource 2, Appendix 1***).

In addition to the finding of negative TTIs, the number of cases from 2015 to 2019 were reducing. There was inadequate supervision of data collection in that period and this led to incompleteness. Efforts to complete data processing for these years was ongoing.

We extracted and provided the list of all the cases with negative TTIs to the registry for checking and possible re-abstraction. We recommended that they consider revising the CanReg5 software, the tool that is used for cancer registration in low-and-middle income countries, to include a flagging mechanism to flag cases whenever treatment dates entered were before incidence dates, so that remedial measures are taken as soon as the cases are identified. This is important because case finding for archived cases can be tedious and resource intensive. Most patient files are paper based and older files are often archived in storage spaces (stores, containers, basements and attics) to create room for current files.

Like we did previously with missing values, to check if the missing treatment start dates occurred at random, we compared the distribution of TTI values for the entire dataset, the negative TTIs and positive TTIs based on variables that are important determinants of TTI or sub groups in the population; age-group, sex, stage at diagnosis and year of diagnosis in the R program ***[R Codes: Resource 2, Section D]***, then used histograms in MS Excel to visualize the data (Figure 3).

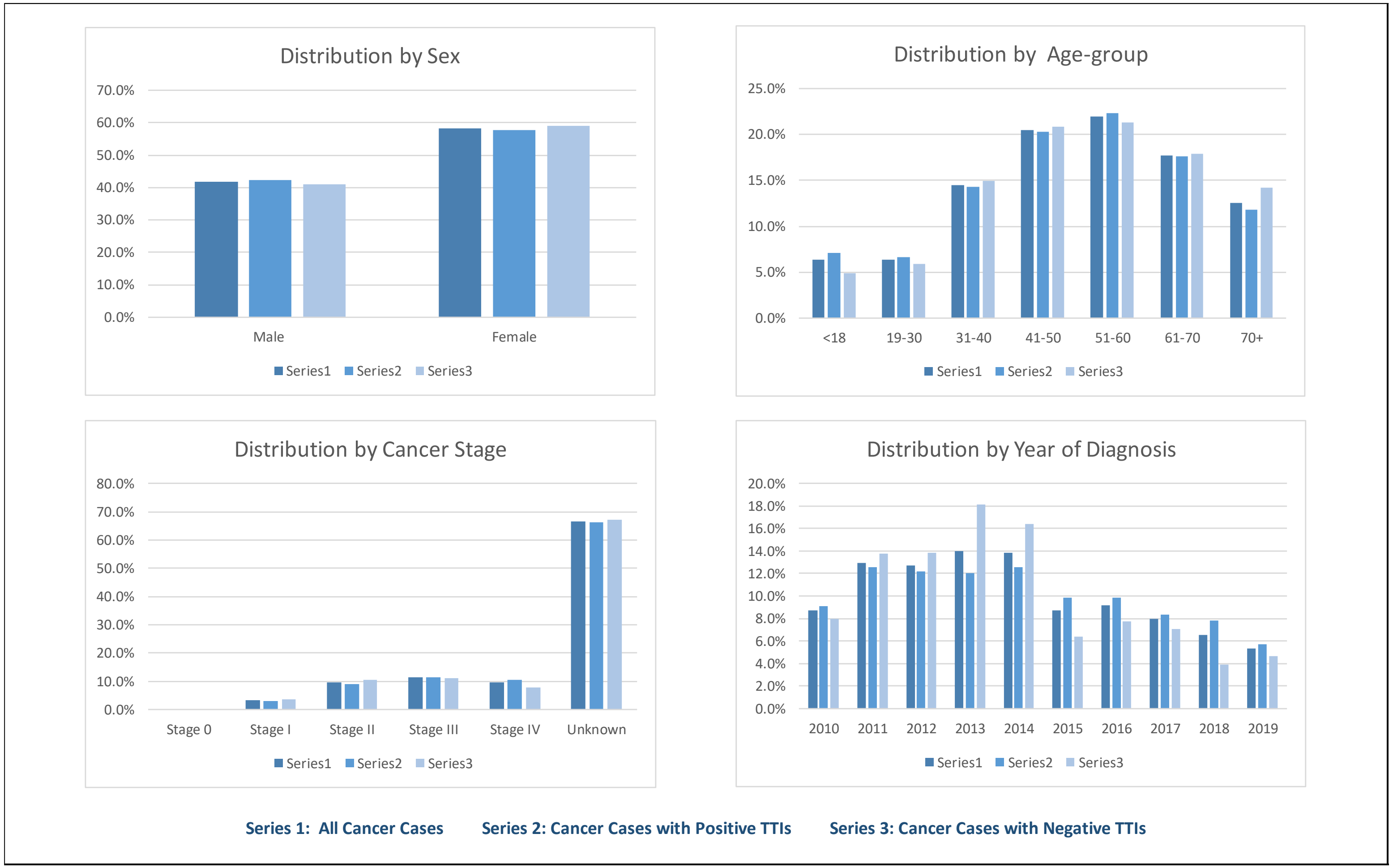

Fig 3. Understanding distribution of cases with and without errors on treatment start dates/incidence dates
Comparing the entire dataset (Series 1), the cases with positive TTI (Series 2) and the cases with negative TTI (Series 3). Comparisons are based on variables that are important determinants of TTI or subgroups in the population; sex, age-group, stage at diagnosis and year of diagnosis. Data from the Nairobi Cancer Registry (2010-2019).

The frequency distributions of TTIs for the entire dataset, positive TTI and negative TTI categories compared reasonably well, hence our decision to exclude all the negative TTI cases from the analysis with the assumption that the analysis of the positive TTI subset would be a good representation of the entire dataset and that the negative TTI values occurred at random. A total of 3551 (31.9%) of the data with at least one treatment date recorded had the negative TTI values. The valid subset (dataset with positive TTIs) had 7548 cases.

We extracted the valid dataset (n=7584) then computed the demographic distributions, the distribution by cancer clinical characteristics and the distribution of cases by year of diagnosis. ***[R Codes:Resource 2, Section E].***

In order to smooth the rough curve in the time series analysis for the cases with positive TTIs (7584 cases) and to remove outliers, we computed the primary outcome measure - monthly median TTI (in days) and obtained 120 observations (aggregated values) in total for our ITSA ***[R Codes: Resource 2, Section F].***

Figure 4 is the flowchart that summarizes the data cleaning and data manipulation process for this study.

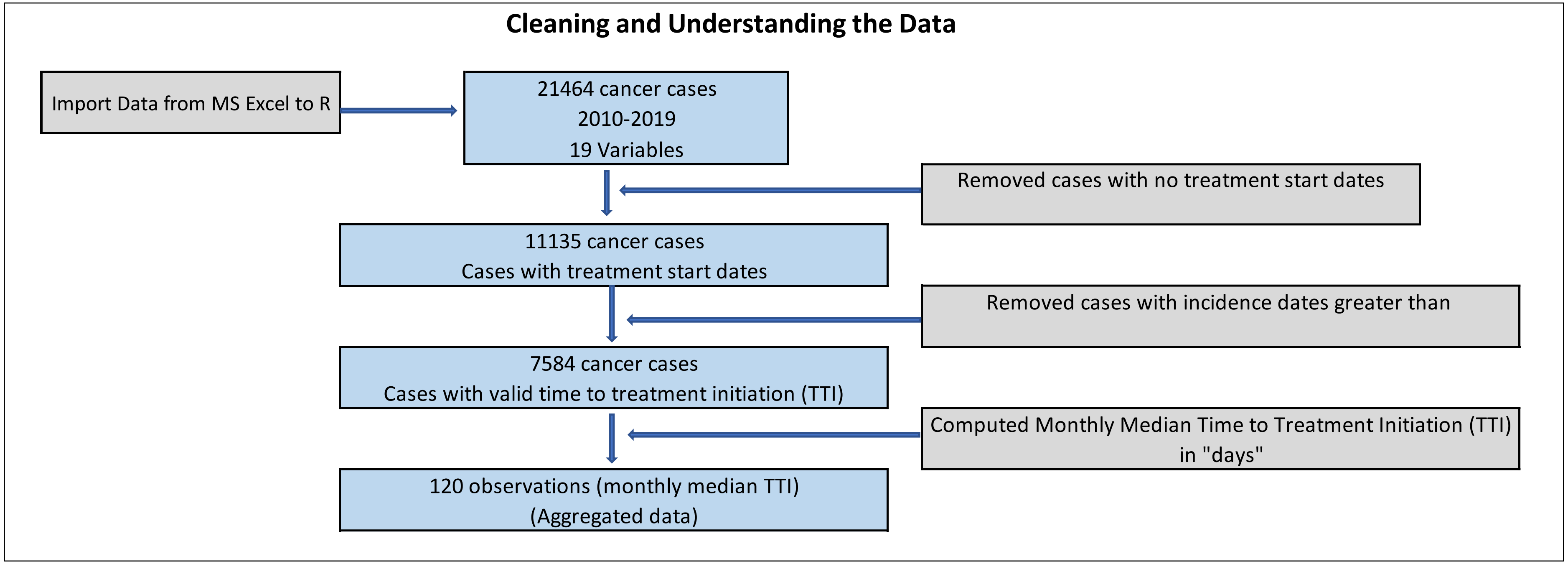

Figure 4. Data Cleaning/Manipulation Process for Interrupted Time Series Analysis.
Nairobi Cancer Registry Data (2010-2019)

R Codes

Resource 2_R Codes; Data Cleaning/Manipulation and Descriptive Analysis

```
#Section A
#Imported data from Excel to R
library(readxl)
CancerData_ITS <- CancerData_2010_2019_Dx_and_treatment_13April2022
#Explored the Data
#All cancer data from the Nairobi Cancer Registry: 2010-2019
#21464 cases
#19 variables: Age, incidence data, basis of diagnosis, primary site,
morphology, grade, stage, surgery, surgery date, radiotherapy, radiotherapy
date, chemotherapy, chemotherapy date, hormonotherapy, hormonotherapy date,
immunotherapy, immunotherapy date, registration number, sex.

#Checked and converted dates to “date” format
#Checked "class" for incidence date
class(CancerData_2010_2019_Dx_and_treatment_13April2022$`incidence date`)
#numeric
#Created a duplicate dataset; CancerData_ITS
CancerData_ITS<-CancerData_2010_2019_Dx_and_treatment_13April2022
#Created new variable "incidence date2" - "date" variable for incidence
date
CancerData_ITS$`incidence date2`<-as.Date(as.character(CancerData_ITS
$`incidence date`),"%Y%m%d")
#Changed dates to date format
CancerData_ITS$SurgeryDate2<-
as.Date(as.character(CancerData_ITS$surgeryDate),"%Y%m%d")
CancerData_ITS$RadioDate2<-
as.Date(as.character(CancerData_ITS$RadioDate),"%Y%m%d")
CancerData_ITS$ChemoDate2<-
as.Date(as.character(CancerData_ITS$ChemoDate),"%Y%m%d")
CancerData_ITS$HomonDate2<-
as.Date(as.character(CancerData_ITS$HomonDate),"%Y%m%d")
CancerData_ITS$ImmunoDate2<-
as.Date(as.character(CancerData_ITS$ImmunoDate),"%Y%m%d")
#Created a variable - incidence year
CancerData_ITS$`incidence year`<-substring(CancerData_ITS$`incidence
date2`,1,4)

#Computed Time to Treatment Initiation – TTI (in days)
# Created variable for earliest Treatment Date (Rx_Start_Date)
library(dplyr)
CancerData_ITS$Rx_Stat_Date <-apply(CancerData_ITS[,c(21:25)],1,min,na.rm =
TRUE)
#Converted Rx_Stat_Date to "date" format
CancerData_ITS$Rx_Stat_Date2<-
as.Date(as.character(CancerData_ITS$Rx_Stat_Date),"%Y%m%d")
#Computed TTI (days)
CancerData_ITS$TTI_Days<-difftime(CancerData_ITS$Rx_Stat_Date,
CancerData_ITS $`incidence date2`, units = "day")
class(CancerData_ITS$TTI_Days)
CancerData_ITS$TTI_Days <- as.numeric(CancerData_ITS$TTI_Days)

#Section B
#Checked the distribution of cases based on selected determinants of TTI
#Exported data to Ms Excel
# Removed “NAs” from variable TTI_Days
```

```
CancerData_ITS<-filter(CancerData_ITS,!is.na(CancerData_ITS$TTI_Days))
#(11135 cases left)

#Section C
#Create histogram to visualize data
hist(CancerData_ITS$TTI_Days,main = "Frequency of Time to Treatment
Initiation for Incident Cancers in Nairobi County, Kenya (2010-2019)", xlab
= "Time to Treatment Initiation (days)", ylab =
"Frequency",las=1,cex.axis=0.5,cex.lab=0.75,cex.main=0.75)
#There are many negatives TTI (days) values

#Section D
#Checked the distribution of cases based on selected determinants of TTI
#Established counts, compare negative and positive values
#Created new variable for +ve and -ve TTI (days)
CancerData_ITS$TTI_status<-
ifelse(CancerData_ITS$TTI_Days<0,"Negative","Positive")
#Counted +ve and -ve TTI (days) values
table(CancerData_ITS$TTI_status)
#Result: -ve 3551 (31.9%), +ve 7584 (68.1%)
#positive TTI only (7584)
CancerData_ITS_pos <- subset(CancerData_ITS, CancerData_ITS$TTI_Days > 0)
#negative TTI only (3551)
CancerData_ITS_neg <- subset(CancerData_ITS, CancerData_ITS$TTI_Days < 0)
#Compare -ve and +ve values in terms of sex, age groups, cancer stage, year
of diagnosis
#by sex
table(CancerData_ITS$Sex) # All cancers: Female 6482, Male 4653
table(CancerData_ITS_pos$Sex) # Male 3201 (42.2%), Female 4383 (57.8%)
table(CancerData_ITS_neg$Sex) # Male 1452 (40.9%), Female 2099(59.1%)

#By age group
#Create age groups
CancerData_ITS$AgeGroups<-
cut(CancerData_ITS$age,breaks=c(0,18,30,40,50,60,70,120),
labels=c("<18","19-30","31-40","41-50","51-60","61-70","70+"))
table(CancerData_ITS$AgeGroups) # All cancers
#<18  19-30      31-40      41-50      51-60      61-70      70+
# 707   708      1612       2276       2443       1969       1398
table(CancerData_ITS_pos$AgeGroups)
#<18  19-30      31-40      41-50      51-60      61-70   70+
# 535 499        1082       1535       1688       1335   893
table(CancerData_ITS_neg$AgeGroups)
#<18  19-30      31-40      41-50      51-60      61-70      70+
#172  209        530        741        755        634        505
#By cancer stage
table(CancerData_ITS$stage)# All cancers
#StageI     StageII     StageIII     StageIV     Unknown
#346        1056        1260         1062        7411
```

```
table(CancerData_ITS_pos$stage)
#StageI     StageII     StageIII    StageIV     Unknown

#222        690         864         782               5026

table(CancerData_ITS_neg$stage)
#StageI     StageII     StageIII    StageIV     Unknown

#124        366         396         280         2385

#By year of diagnosis

table(CancerData_ITS$`incidence year`)
#2010 2011  2012  2013  2014  2015  2016  2017  2018  2019

#973  1440  1417  1560  1538  972   1020  887   730   598

table(CancerData_ITS_pos$`incidence year`)
#2010 2011  2012  2013  2014  2015  2016  2017  2018  2019

#691  950   925   915   955   744   744   637   591   432

table(CancerData_ITS_neg$`incidence year`)
#2010 2011  2012  2013  2014  2015  2016  2017  2018  2019

#282  490   492   645   583   228   276   250   139   166

#Subset Cancer Data_ positive TTI only (7584 cases)

CancerData_valid <- subset(CancerData_ITS, CancerData_ITS$TTI_Days > 0)
#Check values using histogram

hist(CancerData_valid$TTI_Days,main = "Histogram_Valid Cases; Time To
Treatment Initiation", xlab = "TTI (days)", ylab = "Frequency")
```

#Section E

```
#tabulate by type of treatment

table(CancerData_valid$Immunotherapy)
table(CancerData_valid$surgery)
table(CancerData_valid$Chemotherapy)
table(CancerData_valid$Homonetherapy)
table(CancerData_valid$Radiotherapy)
```

Resource 2_R codes; Preparation for Interrupted Time Series Analysis

```
#Section F
#Preparation for ITSA-ARIMA Modelling
class(CancerData_valid)
#Aggregated TTI days into Monthly Median values
CancerData_W <- CancerData_valid    # Duplicate data
# Created year column
CancerData_W$year <- strftime(CancerData_W$`incidence date2`, "%Y")
# Created month column
CancerData_W$month <- strftime(CancerData_W$`incidence date2`, "%m")
#Aggregated data (Monthly Median Values)
CancerData_aggr <- aggregate(TTI_Days ~ month + year,
                             CancerData_W,
                             FUN = median)
#Created a "date" value to represent each month
CancerData_aggr$date <-
as.Date(paste(CancerData_aggr$year,CancerData_aggr$month, "01", sep="-"),
"%Y-%m-%d")
#checked class
class(CancerData_aggr)
#created subset - CancerData_aggr2 (2 columns)
CancerData_aggr2<-CancerData_aggr[c("date","TTI_Days")]
CancerData_aggr2
class(CancerData_aggr2)
```

Appendix 1 –AFCRN standard operating procedures - Information on the tumour

Date of incidence
The date of the first event (of the six listed below) to occur chronologically should be chosen as incidence date. If an event of higher priority occurs within three months of the date initially chosen, the date of the higher priority event should take precedence. Order of declining priority:

1. Date of first histological or cytological confirmation of this malignancy (with the exception of histology or cytology at autopsy). This date should be, in the following order:
   a. a date when the specimen was taken (biopsy)
   b. date of receipt by the pathologist
   c. date of the pathology report

2. Date of admission to the hospital because of this malignancy.

3. When evaluated at an outpatient clinic only: date of first consultation at the outpatient clinic because of this malignancy.

4. Date of diagnosis, other than 1, 2 or 3. It may be date of first clinical investigation procedure fort the malignancy e.g.: MRI reports, CT scan reports etc.

5. Date of death, if no information is available other than the fact that the patient has died because of a malignancy.

6. Date of death, if the malignancy is discovered at autopsy.

Whichever date is selected, the date of incidence should **NOT** be later than the date of the start of the treatment, or decision not to treat, or date of death.

Ref: AFCRN-SOP-Part-I.pdf [Internet]. [cited 2023 May 7]. Available from: http://www.afcrn.org/images/Publication/AFCRN-SOP-Part-I.pdf
