# Supplementary material for: Evaluating the impact of the National Health Insurance Fund oncology benefits package and a healthcare workers’ strike on time to cancer treatment initiation in Nairobi County, Kenya: An interrupted time series analysis
Source: PLoS One. 2025 May 22;20(5):e0324593. doi: 10.1371/journal.pone.0324593 (PMC12097610; doi:10.1371/journal.pone.0324593)
Supplement: S3 Fig — Nairobi County cancer registry data (2010–2019). (PDF) [file pone.0324593.s003.pdf]

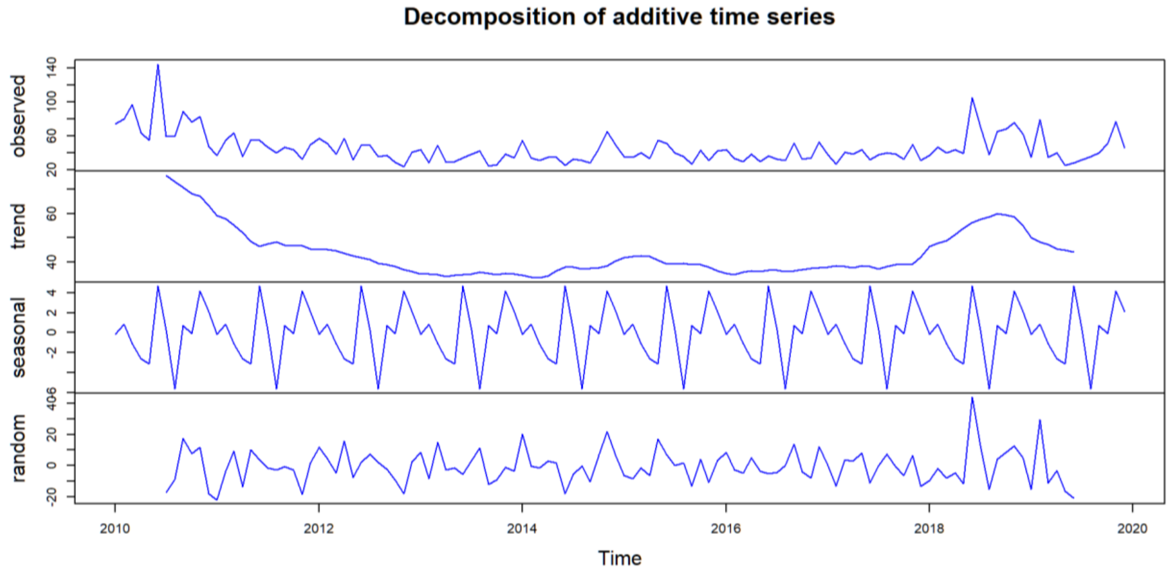

Fig 1. Decomposition of time series of monthly median Time to Treatment Initiation Nairobi County cancer registry data (2010-2019)
