# Supplementary material for: Evaluating the impact of the National Health Insurance Fund oncology benefits package and a healthcare workers’ strike on time to cancer treatment initiation in Nairobi County, Kenya: An interrupted time series analysis
Source: PLoS One. 2025 May 22;20(5):e0324593. doi: 10.1371/journal.pone.0324593 (PMC12097610; doi:10.1371/journal.pone.0324593)
Supplement: S4 File — Details of data analysis and R codes. (PDF) [file pone.0324593.s004.pdf]

S4 Supporting Information

Steps for ARIMA/SARIMA modelling

Interrupted time series analysis (oncology insurance scheme intervention)

Step 1: Visualizing the data to understand patterns

We loaded the required libraries, plotted to visualise the time series graph (Figure 1), then converted the valid dataset to a time series object (Cdata.ts) and viewed. ***[R codes: resource S4, Section A].***

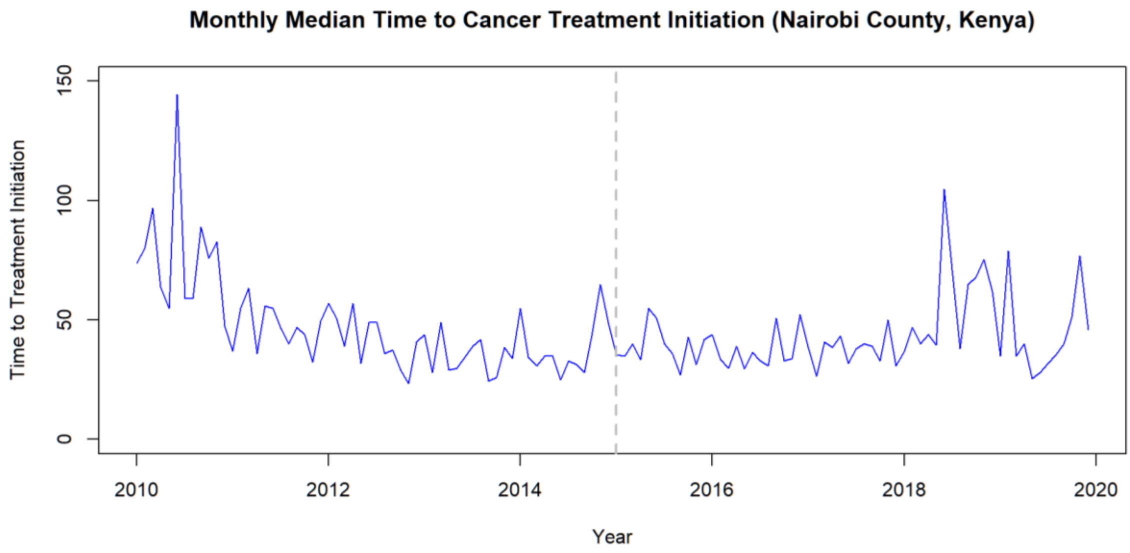

Fig 1. Exploratory time series graph
Monthly median time to cancer treatment initiation for Nairobi County, Kenya; Intervention line - April 2015 NHIF oncology benefits package introduction, Nairobi Cancer Registry Data (2010-2019).

We sought to decompose the time series data (Figure 2) ***[R Codes: resource S4, Section B].***

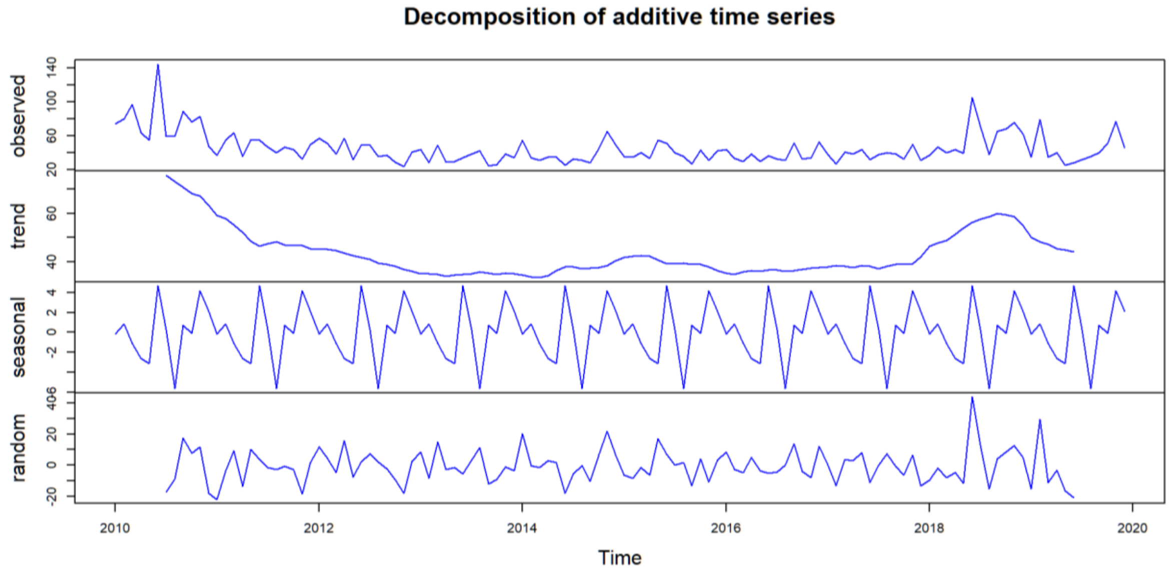

Fig 2. Decomposition of time series of monthly median Time to Treatment Initiation Nairobi County cancer registry data (2010-2019)

Figure 2 shows that there was a general downward trend from 2010 to 2014, a gentle rise to 2015, then a gradual decrease towards 2016, followed by a relatively steep rise in 2018 and a downward trend in 2019. Seasonality patterns were evident.

Step 2: Checking for stationarity

After visualizing the presence of trends and seasonality, we needed to check for stationarity of the time series. Stationarity is a time series property where statistical properties (mean, variance and covariance) do not change with time (1) and is a key assumption in ARIMA /SARIMA modeling. We used the Augmented Dickey-Fuller test on our series in R and got a p-value of 0.4533 ***[R codes: resource S4, Section C].*** The null hypothesis for this test indicates non-stationarity. There was not enough evidence to reject the null hypothesis at 0.05 significance level hence the data was treated as non-stationary. Non stationarity meant that the validity of necessary assumptions for reliability of the ITSA could not hold. Verifying stationarity helps to ensure the validity of the underlying assumptions and the reliability of subsequent analysis and forecasting of the time series data.

Step 3: Checking for autocorrelation

The ACF (Autocorrelation Function) and PACF (Partial Autocorrelation Function) are two important tools used to assess the autocorrelation structure of time series data. Autocorrelation exists when "*observations in a time series are correlated with observations at previous time points hence violating their independent distribution*" (1). We carried out the ACF and PACF analysis to view ACF/PACF plots of the undifferenced data using lag 12 because of the assumption of annual

seasonality in the outcome variable (monthly median TTI) (Figure 3) ***[R codes: Resource S4, Section D].***

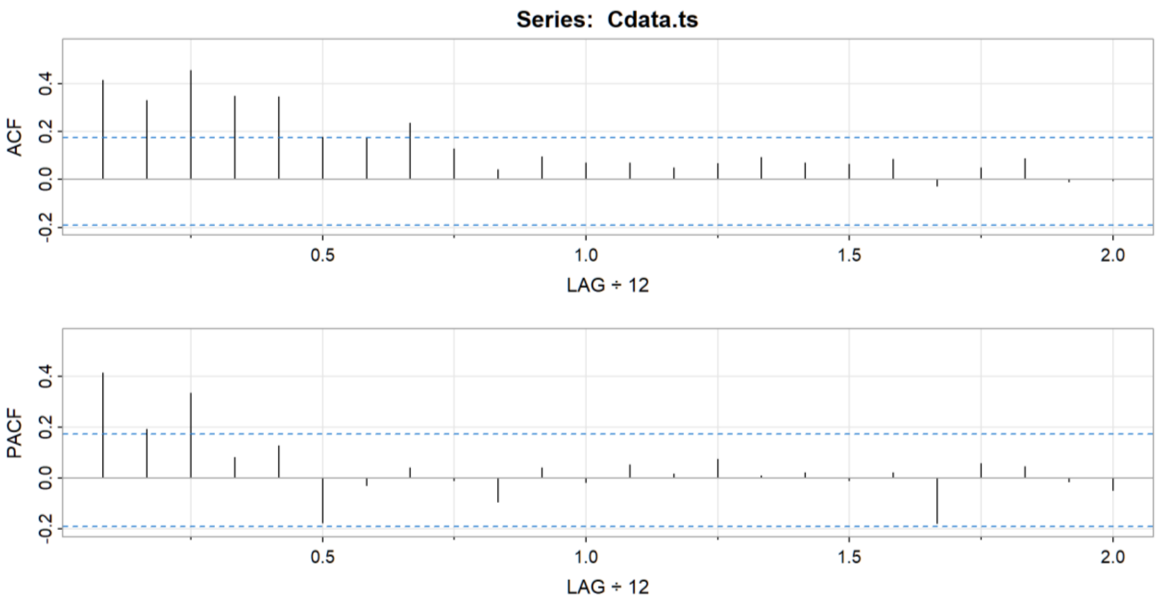

Fig 3. ACF and PACF tests; evaluating the presence of autocorrelation in the monthly median time to treatment initiation
Nairobi cancer registry data (2010-2019).

The lines going below and above the dashed line, and the slow decay of the of the ACF suggest that data were autocorrelated and required differencing before ARIMA/SARIMA modelling.

Step 4: ARIMA/SARIMA Modelling

The choice of using ARIMA/SARIMA modelling for this ITSA was appropriate because the data exhibited complex trends and seasonality. Segmented regression, which is often used, does not control for seasonal effects and trends within the data. Using segmented regression would bias the results (1).

Step 2 demonstrated that the time series data were non-stationary, step 3 demonstrated that the data were autocorrelated and figure 1 and 2 showed seasonal patterns in the data. We applied the *auto.arima ()* function in the *forecast package* of R to identify the best ARIMA model terms for our ITSA. The *auto.arima ()* function in R automates the selection of an ARIMA model based on the provided data. "*The algorithm in auto.arima () uses a stepwise search across the model space to select the best model with smallest Akaike Information Criterion (AIC) or Bayesian Information Criterion (BIC)*" (2). We pre-specified first order differencing of the time series to ("d=1") to control for autocorrelation and to allow for the intervention impact to be estimated. The result was "*Best*

Model: Regression with ARIMA (0,1,4)" and the minimum AIC was 994.3784 ***[R code: resource S4, Section E]***.

The (0,1,4) values correspond to the p,d, and q parameters selected ("p" and "q" automatically) by minimizing the AIC after running the *auto.arima ()* function in the forecast package in R. They define the non-seasonal components of the model. The model, ARIMA (0,1,4), meant that the autoregressive (AR) order (p) was 0, the non-seasonal differencing order (d) was 1 (which we prespecified based on common practice where first differencing is often sufficient (1)), and the moving average (MA) order (q) was 4 (3). The AR order of 0 implies that there was no direct dependence on past observations, which is a reasonable assumption for monthly median TTI in cancer. A differencing order of 1 implies that the data was differenced once to achieve stationarity and the MA order of 4 means that the model included four lagged forecast errors to capture dependencies and fluctuations in the data.

Step 5: The Ljung-Box test; testing if residuals are random and uncorrelated (white noise)

To help ensure appropriateness and accuracy of the model, we performed a Ljung-Box test using the *Box.test()* function in R, a statistical test to check for autocorrelation in the residuals in a time series. Residuals are the deviations between observed values and the predicted ***[R code: resource S4, Section F].***

The null hypothesis in the Ljung-Box test indicates that the residuals of the time series are independently and identically distributed (no significant autocorrelation present). With the p-value of 0.9935 obtained at 0.05 level of significance, there was no strong evidence to reject the null hypothesis, hence the test suggested that the model's residuals were independent and the model would capture the data patterns well. The residuals pattern in the figure did not have an obvious pattern (no autocorrelation) and the histogram with a density curve was of normal distribution with a mean of zero and constant variance, which implied the presence of "white noise" (Figure 4).

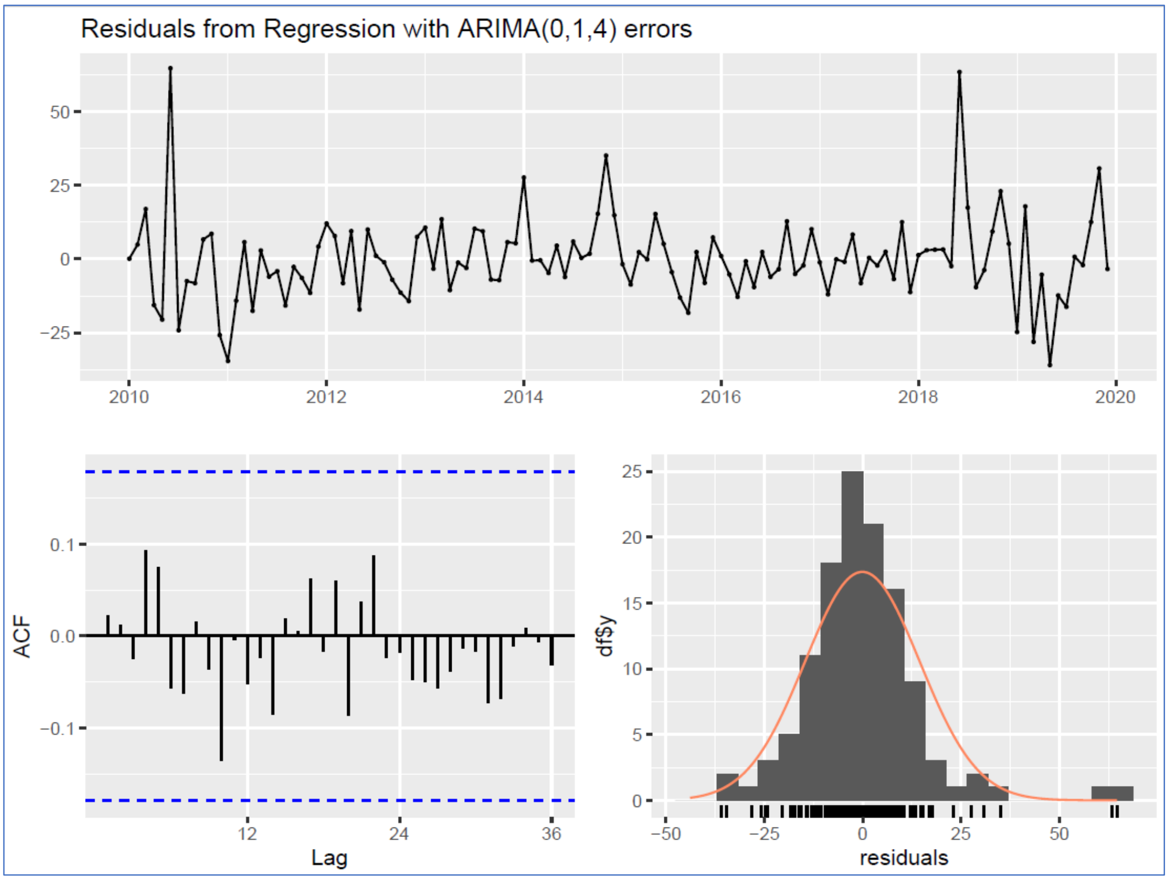

Fig 4. White Test; Residuals check from regression with ARIMA (0,1,4)
Time series - monthly median time to treatment initiation, Nairobi Cancer Registry data (2010-2019)

The white noise displayed in figure 4 means that the model was correctly specified.

Step 6: Including a seasonality component before forecasting the counterfactual

There was need to include a seasonality component before forecasting based on the observation in figure 1 (decomposition of time series) which revealed that the observed data had a seasonality component. We needed to select “P”, “D” and “Q” components for a SARIMA (Seasonal Autoregressive Integrated Moving Average) model - an extension of the ARIMA model. "P" represents the *seasonal autoregressive order*, capturing the seasonal relationship with past observations; "D" represents the *seasonal differencing order*, achieving seasonal stationarity by removing seasonal patterns; and "Q" represents the *seasonal moving average order*, capturing dependencies on past forecast errors at seasonal lags. These parameters define the seasonal components of the SARIMA model and are crucial for capturing and forecasting seasonal patterns in the time series data. We used the *auto.arima()* function in the “*forecast*” package in R to fit the best SARIMA model for the time series dataset.

We chose (0,1,1) in order to include a “D” term of 1 and a “Q” term of 1. Similar to the non-seasonal component description, the seasonal component (0,1,1)(12) meant that the chosen seasonal autoregressive (SAR) order (P) was 0, the seasonal differencing order (D) was 1, and the seasonal moving average (SMA) order (Q) was 1, with a seasonal period of 12. The SAR order of 0 meant no direct dependence on past observations at seasonal intervals. A seasonal differencing order of 1 meant that the seasonal data had been differenced once to achieve seasonal stationarity and an SMA order of 1 meant that the model incorporated one lagged seasonal forecast error to capture seasonal dependencies. The auto generated model with our specified parameters had an AIC of approximately 996 which was not far from the original 994. A lower AIC value indicates a better trade-off between model fit and complexity.

We performed the Ljung-Box test for white noise on the residuals of the model, yielding a p-value of 0.9745. Based on the significance level of 0.05, there was no statistically significant evidence of autocorrelation in the residuals. The residuals from the regression with SARIMA (0,1,4) (0,1,1) (12) were somewhat normally distributed with zero mean and had constant variance and the vertical lines in the ACF plot did not cross the dashed horizontal lines (Figure 5) ***[R code: resource S4, Section G].***

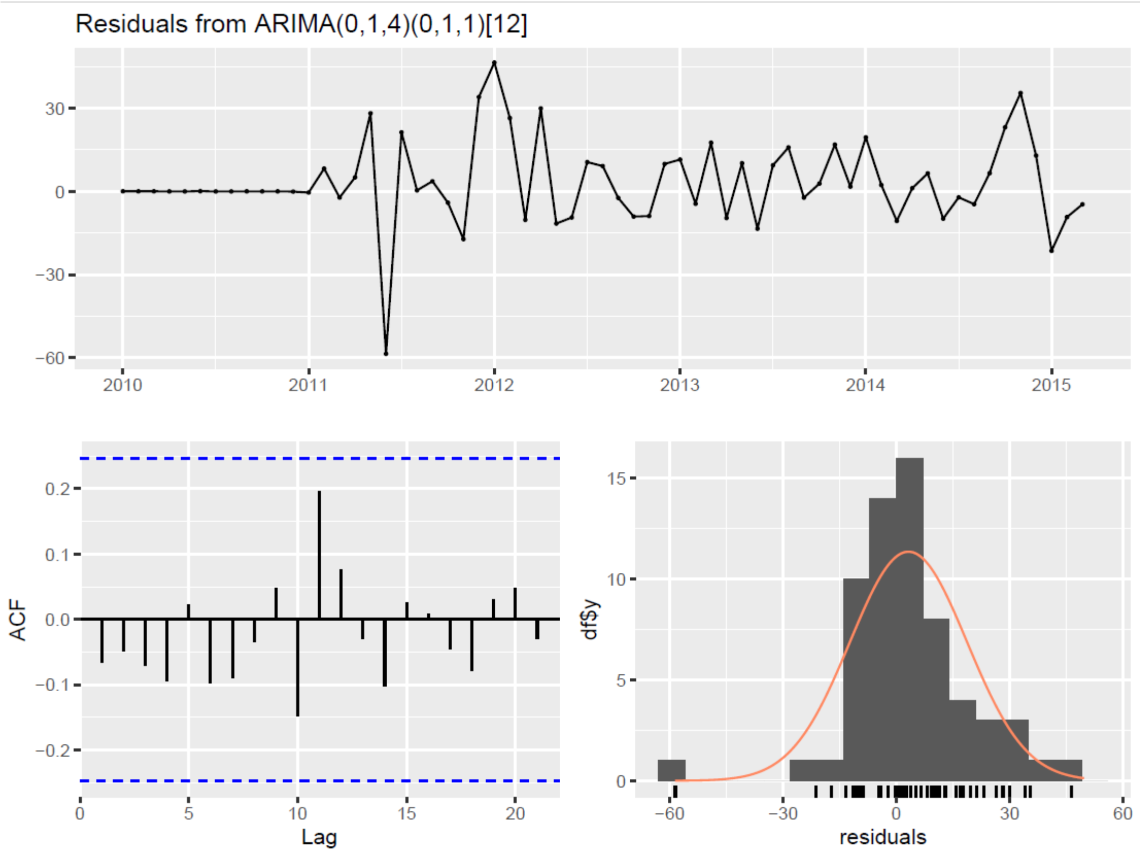

Fig 5. White Test; Residuals check for sARIMA model (0,1,4) (0,1,1) (12)
Time series – monthly median time to treatment initiation, Nairobi Cancer Registry data (2010-2019)

This model was correctly specified and would therefore sufficiently control for autocorrelation and seasonality. It was expected to allow for correct forecasts. With the properly specified models, we forecasted a counterfactual and were able to estimate the regression coefficients and confidence intervals to test for statistical significance of step and ramp changes at the intervention points in the time series.

Step 7: Forecasting the counterfactual

In the absence of a suitable control, we used the identified model (0,1,4)(0,1,1)(12) to forecast a counterfactual to display predicted values against the observed values in the time series. We forecasted a counterfactual series, 57 months post-intervention, converted this to time series object and plotted the graph (Figure 6) ***[R code: resource S4, Section H].***

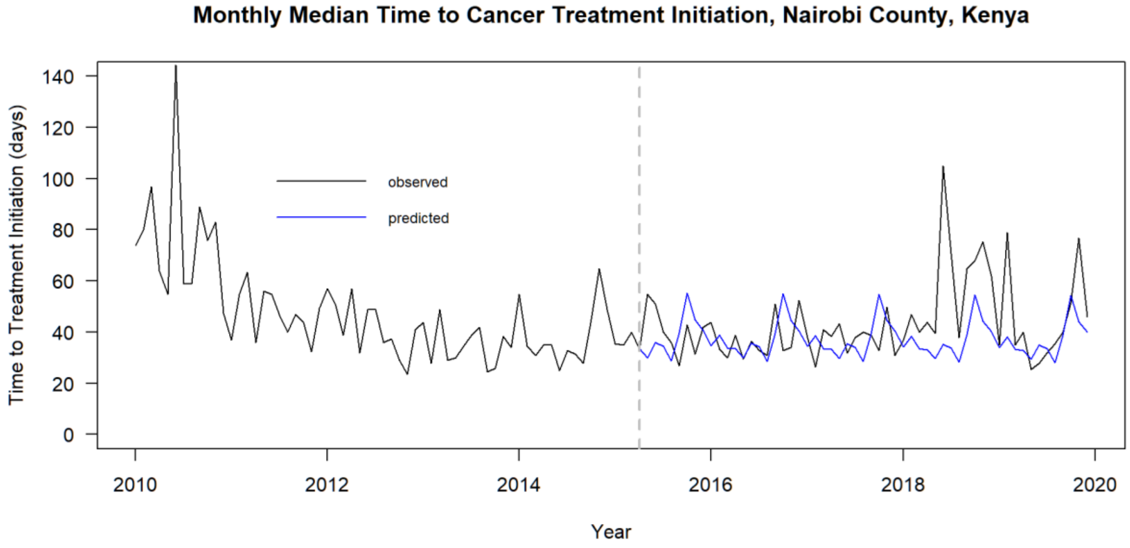

Estimated parameters and confidence intervals for the ramp and step change:

	coefficients	confidence Intervals (95%)	standard errors (s.e)	statistical significance
**ramp**	*0.92*	*-0.12 - 1.86*	*0.48*	*no*
**step**	*7.97*	*-9.92 - 25.86*	*9.13*	*no*

Fig 6. Interrupted Time series Analysis; the National Health Insurance Fund (NHIF) oncology package Intervention (April 2015) for Nairobi County, Kenya.
(2010 -2019 Nairobi Cancer Registry data)

Step 8: Model results for the step and ramp changes

We estimated the regression coefficients and confidence intervals to test for statistical significance of the step and ramp changes arising from the intervention in the time series ***[R code: resource S4, Section I]*** (Figure 6).

Exploring the 2018 increase in the monthly median TTI value

We used the same process that we used for plotting and estimating coefficients for the oncology insurance scheme impact for the 2018 increase in TTI ***[R code: resource S4, Section J]*** (Figure 7)

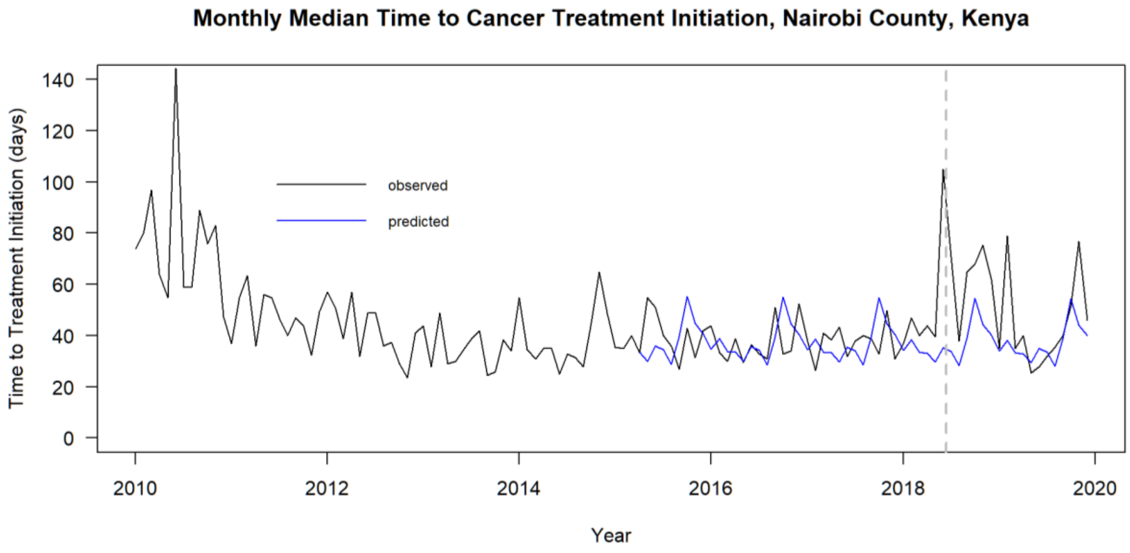

Estimated parameters and confidence intervals for the ramp and step change:

	coefficients	confidence Intervals (95%)	standard errors (s.e)	statistical significance
**ramp**	*-1.6*	*-3.5 –* 0.4	*1.0*	*No*
**step**	*34.6*	*15.4 – 53.8*	*9.8*	*Yes*

Fig 7. Interrupted Time series Analysis: Monthly Median Time to Treatment Initiation the Intervention line in June 2018 for Nairobi County, Kenya.
(2010 -2019 Nairobi Cancer Registry data)

Sensitivity Analysis

Sensitivity analyses assess the internal validity and robustness of findings within a study. They can be tailored to examine the impact of different assumptions, model specifications, data and time frame variations on study results. We analysed the impact of reducing the pre-intervention time frames (hence pre-intervention data points) and the effect of starting the counterfactual closer to the interruption. This was to determine if the estimated effect was robust based on changing numbers of data points before the intervention - (2010-2019 data) versus (2013-2019 data) and whether the counterfactual plot would change if started closer to the interruption. Reducing the number of data points (sample size) is expected to reduce the statistical power of the analysis, elicit wider confidence intervals and less precision in the estimates (4).

Variation of the pre-intervention time frames (the oncology insurance scheme intervention time series)

Figure 8 is the time series plot for the reduced pre-intervention time frame - analysis from the 2013 – 2019 time series subset. We included the 2010-2019 effect parameters for comparison ***[R code: resource S4, Section K].***

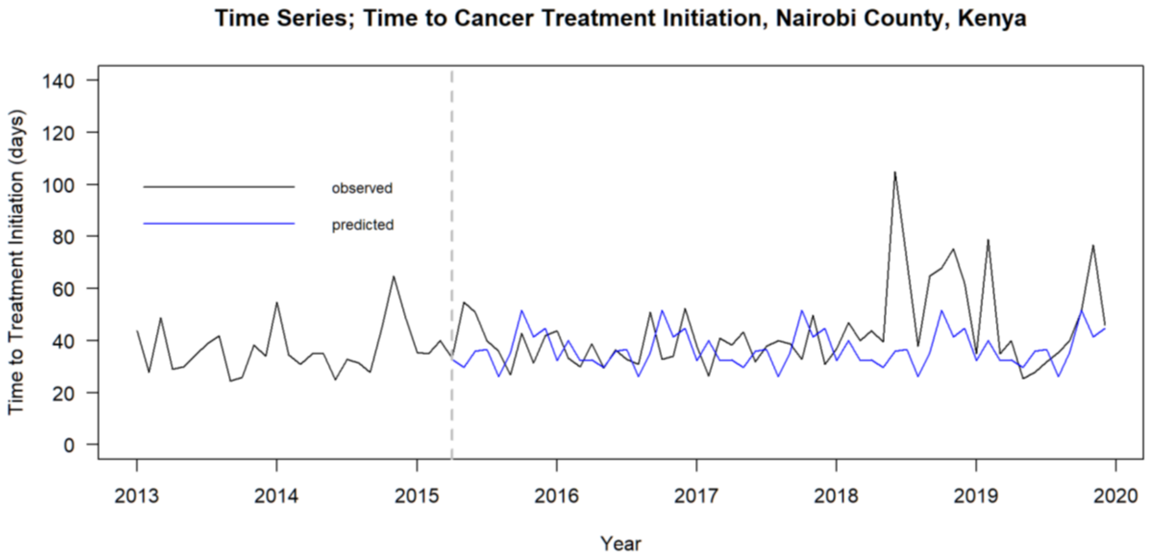

	coefficients	confidence Intervals (95%)	standard errors (s.e)	statistical significance
ramp (2013-2019)	***0.29***	***0.03 – 0.54***	***0.13***	***Yes***
ramp (2010-2019)	*0.92*	*-0.02 - 1.86*	*0.48*	*No*
step (2013-2019)	***-1.56***	***-11.76 – 8.86***	***5.12***	***No***
step (2010-2019)	*7.96*	*-9.92 - 25.86*	*9.13*	*No*

Fig 8. Interrupted Time series Analysis: introduction of the NHIF oncology benefits package (April 2015) - Forecasting counterfactual from March 2015
Nairobi County, Kenya (2013 -2019/2010-2019 Nairobi Cancer Registry data)

The ramp (gradual) change of 0.29 days (95% CI 0.03 to 0.54) per month was statistically significant in the 2013-2019 data but not in the 2010-2019 data. This implies that unlike in the 2010-2019 data, where there was an apparent change, the 2013-2019 data model had a 0.29 day increase per month in monthly median TTI after the introduction of the oncology insurance scheme. Standard errors were smaller with the 2013-2019 data set.

Variation of the pre-intervention time frames; healthcare workers strike interruption time series

Figure 9 is the time series plot for the reduced pre-intervention time frame - analysis from the subset (2013 – 2019 time series data) for the healthcare workers strike interruption. We included the 2010-2019 effect parameters for comparison ***[R code: resource S4, Section L].***

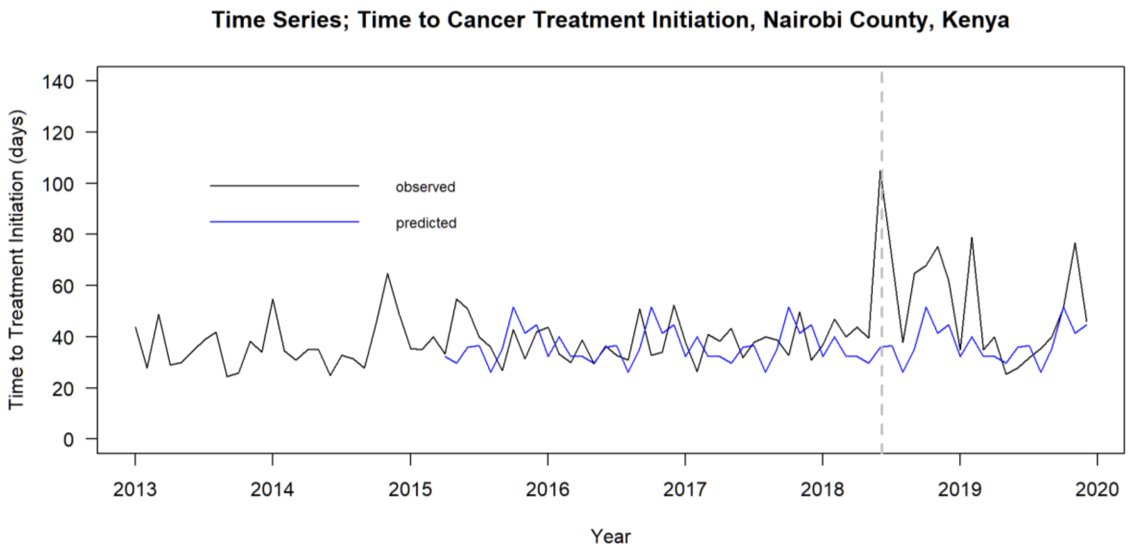

	coefficients	confidence Intervals (95%)	standard errors (s.e)	statistical significance
ramp (2013-2019)	***-1.8***	***-2.8 – -0.9***	***0.5***	***Yes***
ramp (2010-2019)	*-1.6*	*-3.5 – 0.4*	*1.0*	*No*
step (2013-2019)	***33.4***	***22.3 – 44.5***	***5.6***	***Yes***
step (2010-2019)	*34.6*	*15.4 – 53.8*	*9.8*	*Yes*

Fig 9. Interrupted Time series Analysis: healthcare workers strike (2018) for Nairobi County, Kenya – Forecasting counterfactual from March 2015
(2013 -2019/2010-2019 Nairobi Cancer Registry data)

The ramp change was statistically significant in 2013-2019 data; -1.8 (95% CI -2.8 to -0.9) but not in the 2010-2019 data. The step changes are both statistically significant at an approximate increase of 33 to 34 days monthly median TTI (Figure 9). This could mean that the strike had an immediate large impact (step change) followed by gradual reduction over time (ramp change) of 1.8 days per month for the 2013-2019 data and no slope change for 2010-2019 data. Standard errors were smaller with the 2013-2019 data set.

References

1. Schaffer AL, Dobbins TA, Pearson SA. Interrupted time series analysis using autoregressive integrated moving average (ARIMA) models: a guide for evaluating large-scale health interventions. BMC Med Res Methodol. 2021 Mar 22;21(1):58.

2. Losada L. Medium. 2020 [cited 2023 Jun 19]. Time Series Analysis with Auto.Arima in R. Available from: https://towardsdatascience.com/time-series-analysis-with-auto-arima-in-r-2b220b20e8ab

3. Li X, Zhang X. A comparative study of statistical and machine learning models on carbon dioxide emissions prediction of China [Internet]. In Review; 2023 Jul [cited 2023 Aug 3]. Available from: https://www.researchsquare.com/article/rs-3070359/v1

4. Zhang F, Wagner AK, Ross-Degnan D. Simulation-based power calculation for designing interrupted time series analyses of health policy interventions. J Clin Epidemiol. 2011 Nov;64(11):1252–61.

Resource S4 – R Codes

Resource S4_R codes; Interrupted Time Series Analysis, ARIMA/SARIMA Modelling _ April 2015 interruption

```
#Interrupted Time series analysis-ARIMA modelling
#Section A
#Loaded required libraries
library(astsa)
library(forecast)
library(dplyr)
library(zoo)
library(ggplot2)
library(tseries)
#Created a copy of the dataset (CancerData_aggr2)
Cdata_ts<-CancerData_aggr2
# Ploted data to visualise time series
options(scipen=5)
plot(Cdata_ts, ylim=c(0,140), type='l', las=1, col="blue", xlab="Year",
ylab="Time to Treatment Initiation (days)", main="Time Series; Time to
Cancer Treatment Initiation, Nairobi County, Kenya")
# Converted data to time series object and viewed
Cdata.ts <- ts(Cdata_ts[,2], frequency=12, start=c(2010,1))
Cdata.ts

#Section B
#Decomposed time series
components.ts = decompose(Cdata.ts)
plot(components.ts,col="blue")

#Section C
#Augmented Dickey-Fuller test
adf.test(Cdata.ts) # p-value = 0.4533

#Section D
# Viewed ACF/PACF plots of undifferenced data
acf2(Cdata.ts, max.lag=24)

#Section E
# Created variable representing step change and viewed
step <- as.numeric(as.yearmon(time(Cdata.ts))>='April 2015')
step
```

```
# Create variable representing ramp (change in slope) and viewed
ramp <- append(rep(0,64), seq(1,56,1))
ramp

# Used automated algorithm to identify p/q parameters
# Specified first difference = 1 and seasonal difference = 1
model1 <- auto.arima(Cdata.ts, seasonal=TRUE, xreg=cbind(step,ramp),
max.d=1, max.D=1, stepwise=FALSE, trace=TRUE)# Best Model: Regression with
ARIMA (0,1,4)

#Section F
# Checked residuals
checkresiduals(model1)
Box.test(model1$residuals, lag = 24, type = "Ljung-Box") # p-value = 0.9935

#Section G
#Incorporating the seasonal component before forecasting the
counterfactual, then checked residuals
model2 <- Arima(window(Cdata.ts, end=c(2015,4)), order=c(0,1,4),
seasonal=list(order=c(0,1,1), period=12))
checkresiduals(model2)
Box.test(model2$residuals, lag = 24, type = "Ljung-Box") # p-value = 0.9745

#Section H
# Forecast 57 months post-intervention and convert to time series object
fc <- forecast(model2, h=57)
fc.ts <- ts(as.numeric(fc$mean), start=c(2015,4), frequency=12)

# Combine with observed data
Cdata.ts.2 <- ts.union(Cdata.ts, fc.ts)
Cdata.ts.2

#Plot
plot(Cdata.ts.2, type="l", las=1, plot.type="s",
col=c('black','blue'),xlab="Year", ylab="Time to Treatment Initiation
(days)",main="Median Monthly Time to Cancer Treatment Initiation, Nairobi
County, Kenya", linetype=c("solid","dashed"), ylim=c(0,140))

# Add vertical line indicating date of intervention (Apr, 2015)
abline(v=2015.25, col="gray", lty="dashed", lwd=2)
```

```
legend(x=2011,y=120, legend=c("observed","predicted"),x.intersp=0.5,
y.intersp=0.5,lty=1,col=c('black','blue'),bty="n",lwd=1.25,cex=0.75)

#Section I
# Estimate parameters and confidence intervals and test for significance of
level and ramp changes
summary(model1)
confint(model1)
#step: 7.9664 (-9.9241 - 25.8568)
#ramp: 0.9237 (-0.0175 - 1.8649)
```

Resource S4_R codes; Interrupted Time Series Analysis, ARIMA/SARIMA Modelling _ June 2018 interruption

```
#Section J
Cdata_ts<-CancerData_aggr2

# Ploted data to visualise time series
options(scipen=5)

# Converted data to time series object and viewed
Cdata.ts <- ts(Cdata_ts[,2], frequency=12, start=c(2010,1))

Cdata.ts

# Created variable representing step change and view
step <- as.numeric(as.yearmon(time(Cdata.ts))>='June 2018')

step

# Created variable representing ramp (change in slope) and viewed
ramp <- append(rep(0,102), seq(1,18,1))

ramp

# Used automated algorithm to identify p/q parameters
# Specified first difference = 1 and seasonal difference = 1
model1 <- auto.arima(Cdata.ts, seasonal=TRUE, xreg=cbind(step,ramp),
max.d=1, max.D=1, stepwise=FALSE, trace=TRUE) # Best model: Regression with
ARIMA(1,1,3)

# Check residuals
checkresiduals(model1)

Box.test(model1$residuals, lag = 24, type = "Ljung-Box") # p - value:9819

# To forecast the counterfactual, from the same time as that used in the
earlier model (April 2015)
model2 <- Arima(window(Cdata.ts, end=c(2015,4)), order=c(1,1,3),
seasonal=list(order=c(0,1,1), period=12))

# Checked residuals
checkresiduals(model2)

Box.test(model2$residuals, lag = 24, type = "Ljung-Box")# p value 0.9878

# Estimate parameters and confidence intervals and test for significance of
level and ramp changes
summary(model1)

confint(model1)

#step: 34.6(15.4 - 53.8)

#ramp: -1.6 (-3.5 - 0.4)

# Forecasted 57 months post-intervention and converted to time series
object
fc <- forecast(model2, h=57)

fc.ts <- ts(as.numeric(fc$mean), start=c(2015,4), frequency=12)

# Combined with observed data
```

```
Cdata.ts.2 <- ts.union(Cdata.ts, fc.ts)

Cdata.ts.2


#Ploted
plot(Cdata.ts.2, type="l", las=1, plot.type="s",
col=c('black','blue'),xlab="Year", ylab="Time to Treatment Initiation
(days)",main=" Monthly Median Time to Cancer Treatment Initiation, Nairobi
County, Kenya", linetype=c("solid","dashed"), ylim=c(0,140))

# Added vertical line indicating interruption (Jun, 2018)
abline(v=2018.45, col="gray", lty="dashed", lwd=2)

legend(x=2011,y=120, legend=c("observed","predicted"),x.intersp=0.5,
y.intersp=0.5,lty=1,col=c('black','blue'),bty="n",lwd=1.25,cex=0.75)
```

Resource S4_R codes; Sensitivity Analysis

```
#Sensitivity Analysis
#Created a subset, 2013-2019 dataset (sub_Cdata_ts)
sub_Cdata_ts <- subset(Cdata_ts, date >= "2013-01-01" & date <= "2019-12-01")
#Section K
Cdata_ts<-sub_Cdata_ts
# Ploted data to visualise time series
options(scipen=5)
plot(Cdata_ts, ylim=c(0,140), type='l', las=1, col="blue", xlab="Year", ylab="Time to Treatment Initiation (days)", main="Time Series; Time to Cancer Treatment Initiation, Nairobi County, Kenya")
# Converted data to time series object
Cdata.ts <- ts(Cdata_ts[,2], frequency=12, start=c(2013,1))
Cdata.ts
#Decomposed time series
components.ts = decompose(Cdata.ts)
plot(components.ts,col="blue")
# Viewed ACF/PACF plots of undifferenced data
acf2(Cdata.ts, max.lag=24)
#Augmented Dickey-Fuller test
adf.test(Cdata.ts)#p-value: 0.1854
# Created variable representing step change and viewed
step <- as.numeric(as.yearmon(time(Cdata.ts))>='Apr 2015')
step
# Created variable representing ramp (change in slope) and viewed
ramp <- append(rep(0,28), seq(1,56,1))
ramp
# Used automated algorithm to identify p/q parameters
# Specified first difference = 1 and seasonal difference = 1
model1 <- auto.arima(Cdata.ts, seasonal=TRUE, xreg=cbind(step,ramp), max.d=1, max.D=1, stepwise=FALSE, trace=TRUE)# Best model: Regression with ARIMA(1,0,0)
# Checked residuals
checkresiduals(model1)
Box.test(model1$residuals, lag = 24, type = "Ljung-Box") #p-value:0.8113
# Estimated parameters and confidence intervals and test for significance of level and ramp changes
summary(model1)
confint(model1)
```

```
# Forecasting the counterfactual; modelled data excluding post-intervention
time period
model2 <- Arima(window(Cdata.ts, end=c(2015,4)), order=c(1,0,0),
seasonal=list(order=c(0,1,1), period=12))
# Checked residuals
checkresiduals(model2)
Box.test(model2$residuals, lag = 24, type = "Ljung-Box")#p-value:9948
# Forecasted 57 months; one month pre-intervention and converted to time
series object
fc <- forecast(model2, h=57)
fc.ts <- ts(as.numeric(fc$mean), start=c(2015,3), frequency=12)
# Combine with observed data
Cdata.ts.2 <- ts.union(Cdata.ts, fc.ts)
Cdata.ts.2
#Plot
plot(Cdata.ts.2, type="l", las=1, plot.type="s",
col=c('black','blue'),xlab="Year", ylab="Time to Treatment Initiation
(days)",main="Time Series; Time to Cancer Treatment Initiation, Nairobi
County, Kenya", linetype=c("solid","dashed"), ylim=c(0,140))
# Add vertical line indicating date of intervention (Apr, 2015)
abline(v=2015.25, col="gray", lty="dashed", lwd=2)
legend(x=2012.5,y=120, legend=c("observed","predicted"),x.intersp=0.5,
y.intersp=0.5,lty=1,col=c('black','blue'),bty="n",lwd=1.25,cex=0.75)

#Section L
Cdata_ts<-sub_Cdata_ts
# Plot data to visualise time series
options(scipen=5)
plot(Cdata_ts, ylim=c(0,140), type='l', las=1, col="blue", xlab="Year",
ylab="Time to Treatment Initiation (days)", main="Time Series; Time to
Cancer Treatment Initiation, Nairobi County, Kenya")
# Convert data to time series object
Cdata.ts <- ts(Cdata_ts[,2], frequency=12, start=c(2013,1))
Cdata.ts
#Decompose time series
library(tseries)
components.ts = decompose(Cdata.ts)
plot(components.ts,col="blue")
# View ACF/PACF plots of undifferenced data
acf2(Cdata.ts, max.lag=24)
#Augmented Dickey-Fuller test
```

```
adf.test(Cdata.ts) #p-value:0.1854

# Create variable representing step change and view
step <- as.numeric(as.yearmon(time(Cdata.ts))>='Jun 2018')
step
# Create variable representing ramp (change in slope) and view
ramp <- append(rep(0,65), seq(1,19,1))
ramp
# Use automated algorithm to identify p/q parameters
# Specify first difference = 1 and seasonal difference = 1
model1 <- auto.arima(Cdata.ts, seasonal=TRUE, xreg=cbind(step,ramp),
max.d=1, max.D=1, stepwise=FALSE, trace=TRUE)# Best model: Regression with
ARIMA(0,0,0)
# Check residuals
checkresiduals(model1)
Box.test(model1$residuals, lag = 24, type = "Ljung-Box") #p-value = 0.9216
# Estimated parameters and confidence intervals and test for significance
of level and ramp changes
summary(model1)
confint(model1)
#step:33.4170 (22.3113-44.5227)
#ramp:-1.8158 (-2.7585--0.8730)
```
