# Supplementary material for: Evaluating the impact of the National Health Insurance Fund oncology benefits package and a healthcare workers’ strike on time to cancer treatment initiation in Nairobi County, Kenya: An interrupted time series analysis
Source: PLoS One. 2025 May 22;20(5):e0324593. doi: 10.1371/journal.pone.0324593 (PMC12097610; doi:10.1371/journal.pone.0324593)
Supplement: S5 File — R codes for model power estimation. (PDF) [file pone.0324593.s005.pdf]

S5 Supporting Information

R codes for model power estimation (SARIMA (0,1,4) (0,1,1) (12)

```
#duplicate the time series data
CancerData_aggr3<-CancerData_aggr2
class(CancerData_aggr3)

#convert to time series data
CancerData_Pts <- ts(CancerData_aggr3$date, frequency = 12, start = c(2010,
1))
pre_intervention_data <- window(CancerData_Pts, end = c(2015, 4))


library(forecast)
# Fit your SARIMA model to pre-intervention data
sarima_model <- Arima(pre_intervention_data,
                      order = c(0,1,4),
                      seasonal = list(order = c(0,1,1), period = 12))  #
period = 12 is inside seasonal


# Forecast the counterfactual (expected trajectory without intervention)
horizon <- length(CancerData_Pts) - length(pre_intervention_data)
counterfactual_forecast <- forecast(sarima_model, h = horizon)

class(pre_intervention_data)

set.seed(42)
n_simulations <- 1000
effect_size <- 7  # Expected deviation from counterfactual
p_values <- numeric(n_simulations)

for (i in 1:n_simulations) {
  # Simulate under the null hypothesis (no intervention)
  simulated_data <- simulate(sarima_model, nsim = horizon)

  # Add intervention effect
  simulated_data <- simulated_data + rnorm(horizon, mean = effect_size, sd
= sd(residuals))

  # Compute residuals for this simulated dataset
  simulated_residuals <- simulated_data - counterfactual_forecast$mean

  # Conduct t-test for each simulation
  p_values[i] <- t.test(simulated_residuals, mu = 0, alternative =
"two.sided")$p.value
}

# Compute statistical power
power_estimate <- mean(p_values < 0.05)
cat("Estimated Power:", round(power_estimate, 2))

# Estimated Power: 0.71
```
